# Supplementary material for: Comparison at the first prenatal visit of the maternal dietary intakes of smokers with non-smokers in a large maternity hospital: a cross-sectional study
Source: BMJ Open. 2018 Jul 12;8(7):e021721. doi: 10.1136/bmjopen-2018-021721 (PMC6082475; doi:10.1136/bmjopen-2018-021721)
Supplement: Supplementary file 1 [file bmjopen-2018-021721supp001.pdf]

Supplementary Table 1:

Comparison of macronutrient intake between never smokers and former smokers

	Never smokers (n=193) (median (IQ range))	Former smokers (n=155) (median (IQ range))	P
Carbohydrate (g)	199.0 (68.2)	197.0 (89.6)	0.535
Protein (g)	72.8 (26.0)	77.0 (29.1)	0.027
Fat (g)	68.8 (40.1)	77.8 (36.4)	0.071
Saturated fat (g)	25.6 (14.2)	28.5 (16.3)	0.211
Monounsaturated fat (g)	24.0 (14.2)	25.5 (12.8)	0.256
Polyunsaturated fat (g)	10.1(7.2)	10.7 (7.3)	0.179
Fibre (g)	16.3 (8.8)	16.2 (8.0)	0.574

(Significance level, 0.05. Statistical test used – Kruskal-Wallis)

IQ range – interquartile range, g – grams

Supplementary Table 2: Comparison of micronutrient intake between never smokers and former smokers

	Never smokers (n=193) (median (IQ range))	Former smokers (n=155) (median (IQ range))	P
Sodium (mg)	1805.2 (928.7)	2069.6 (988.1)	0.008
Potassium (mg)	2472.9 (946.4)	2631.2 (1127.0)	0.102
Chloride (mg)	2688.6 (1294.4)	3067.6 (1329.7)	0.045
Calcium (mg)	749.0 (505.8)	838.0 (533.0)	0.120
Phosphorous (mg)	1124.5 (461.6)	1212.2 (463.5)	0.055
Iron (mg)	9.1 (3.5)	10.0 (5.5)	0.014
Magnesium (mg)	225.1 (98.0)	229.8 (110.3)	0.334
Zinc (mg)	7.7 (3.8)	8.1 (3.9)	0.041
Copper (mg)	0.9 (0.5)	0.9 (0.5)	0.501
Iodine (mcg)	101.8 (69.8)	106.9 (86.7)	0.080
Retinol (mcg)	274.6 (248.2)	310.9 (231.8)	0.089
Carotene (mcg)	3475.6 (5193.8)	2965.4 (4199.2)	0.300
Vitamin C (mg)	79.9 (88.5)	89.5 (78.2)	0.174
Vitamin D (mcg)	2.2 (2.5)	2.5 (2.9)	0.438
Vitamin E (mcg)	8.0 (5.5)	8.4 (5.5)	0.123

Thiamine B1 (mg)	1.4 (0.7)	1.4 (0.6)	0.293
Riboflavin B2 (mg)	1.4 (0.7)	1.6 (0.8)	0.008
Niacin B3 (mg)	33.3 (14.1)	35.8 (15.0)	0.038
Vitamin B6 (mg)	1.9 (0.8)	1.9 (0.8)	0.191
Vitamin B12 (mg)	3.8 (2.8)	4.1 (2.6)	0.018
Folic Acid (mcg)	234.1 (135.9)	249.0 (148.9)	0.154

(Significance level, 0.05. Statistical test used – Kruskal-Wallis)

IQ – interquartile range, mg – milligrams, mcg - micrograms

Supplementary Table 3: Comparison of macronutrient intakes between smokers and non-smokers (excluding likely energy under-reporters i.e. plausible reporters only)

	Non-smokers (n=222) (median (IQ range))[a]	Current smokers (n=34) (median (IQ range))	P
Carbohydrate (g)	217.3 (72.8)	230.0 (94.2)	0.229
Protein (g)	80.2 (25.1)	78.3 (33.5)	0.566
Fat (g)	84.3 (33.0)	90.6 (34.8)	0.416
Fibre (g)	18.3 (8.1)	14.6 (6.3)	0.004
Saturated fat (g)	31.5 (16.6)	35.5 (13.4)	0.472
Monounsaturated fat (g)	29.0 (12.5)	27.9 (12.8)	0.978
Polyunsaturated fat (g)	12.1 (7.3)	10.8 (8.3)	0.236

(Significance level, 0.05. Statistical test used – Kruskal-Wallis)

[a] As dietary composition was similar between never smokers and former smokers (Supplementary tables 1 and 2) they are combined into one "non-smokers" group.

IQ range – interquartile range, g - grams

Supplementary Table 4: Comparison of micronutrient intakes between smokers and non-smokers (excluding likely energy under-reporters i.e. plausible reporters only)

	Non-smokers (n=222) (median (IQ range))[a]	Current smokers (n=34) (median (IQ range))	P
Sodium (mg)	2126.2 (991.6)	2499.4 (1092.7)	0.079
Potassium (mg)	2785.8 (965.7)	2764.8 (1037.3)	0.631
Chloride (mg)	3156.8 (1399.0)	3624.4 (1747.9)	0.031
Calcium (mg)	961.9 (589.9)	848.8 (393.2)	0.396
Phosphorous (mg)	1299.2 (451.4)	1137.7 (479.4)	0.113
Iron (mg)	10.4 (4.3)	9.7 (3.7)	0.023
Magnesium (mg)	252.4 (101.0)	214.7 (80.8)	0.012
Zinc (mg)	8.6 (3.4)	8.1 (3.8)	0.357
Copper (mg)	1.0 (0.5)	0.8 (0.3)	0.009
Iodine (mcg)	120.5 (84.9)	124.8 (92.1)	0.889
Retinol (mcg)	344.9 (282.7)	344.2 (248.9)	0.836
Carotene (mcg)	3213.2 (4762.3)	1488.3 (3572.3)	0.016
Vitamin C (mg)	97.0 (90.8)	77.7 (69.6)	0.097
Vitamin D (mcg)	2.6 (2.9)	2.7 (2.5)	0.726
Vitamin E (mcg)	9.2 (5.4)	8.6 (7.2)	0.321

Thiamine B1 (mg)	1.5 (0.6)	1.5 (0.6)	0.486
Riboflavin B2 (mg)	1.7 (0.8)	1.5 (1.0)	0.244
Niacin B3 (mg)	36.1 (13.7)	33.4 (15.7)	0.562
Vitamin B6 (mg)	2.0 (0.8)	2.1 (0.8)	0.645
Vitamin B12 (mg)	4.5 (2.7)	5.1 (3.9)	0.528
Folic Acid (mcg)	266.2 (123.8)	213.4 (135.9)	0.009

(Significance level, 0.05. Statistical test used – Kruskal-Wallis)

[a] As dietary composition was similar between never smokers and former smokers (Supplementary tables 1 and 2) they are combined into one "non-smokers" group.

IQ range – interquartile range, mg – milligramsmcg – micrograms
