# Supplementary material for: Comparison at the first prenatal visit of the maternal dietary intakes of smokers with non-smokers in a large maternity hospital: a cross-sectional study
Source: BMJ Open. 2018 Jul 12;8(7):e021721. doi: 10.1136/bmjopen-2018-021721 (PMC6082475; doi:10.1136/bmjopen-2018-021721)
Supplement: Author's manuscript [file bmjopen-2018-021721.draft_revisions.pdf]

BMJ Open is committed to open peer review. As part of this commitment we make the peer review history of every article we publish publicly available.

When an article is published we post the peer reviewers' comments and the authors' responses online. We also post the versions of the paper that were used during peer review. These are the versions that the peer review comments apply to.

The versions of the paper that follow are the versions that were submitted during the peer review process. They are not the versions of record or the final published versions. They should not be cited or distributed as the published version of this manuscript.

BMJ Open is an open access journal and the full, final, typeset and author-corrected version of record of the manuscript is available on our site with no access controls, subscription charges or pay-per-view fees (http://bmjopen.bmj.com).

If you have any questions on BMJ Open's open peer review process please email info.bmjopen@bmj.com

BMJ Open

Comparison of the maternal dietary intakes of smokers with non-smokers at the first prenatal visit

Journal:	*BMJ Open*
Manuscript ID	bmjopen-2018-021721
Article Type:	Research
Date Submitted by the Author:	16-Jan-2018
Complete List of Authors:	O'Malley, Eimer<br>Cawley, Shona; University College Dublin, UCD Centre for Human Reproduction; Dublin Institute of Technology<br>Reynolds, Ciara; University College Dublin, UCD Centre for Human Reproduction<br>Kennedy, Rachel; University College Dublin, UCD Centre for Human Reproduction; Dublin Institute of Technology<br>Molloy, Anne; University of Dublin Trinity College, School of Medicine<br>Turner, Michael; University College Dublin
Keywords:	Fetal medicine < OBSTETRICS, Maternal medicine < OBSTETRICS, PUBLIC HEALTH

SCHOLARONE™
Manuscripts

For peer review only

Title: Comparison of the maternal dietary intakes of smokers with non-smokers at the first prenatal visit

Authors: O'Malley EG[1], Cawley S[1,2], Reynolds CME[1], Kennedy RAK[1,2], Molloy A[3], Turner MJ[1].

Affiliations [1]UCD Centre for Human Reproduction, Coombe Women and Infants University Hospital, Cork St, Dublin 8, Ireland

[2] School of Biological Sciences, Dublin Institute of Technology, Kevin St., Dublin 8, Ireland

[3] School of Medicine, Trinity College Dublin, Dublin 2, Ireland.

Corresponding Author: E O'Malley

Email: eimer.om@gmail.com

Telephone: +35314085571

Key words: Maternal dietary intakes, Maternal smoking, Intrauterine fetal growth, Smoking cessation

Word Count: 2927 words

For peer review only

ABSTRACT

- **objectives:** Using detailed dietary and supplement questionnaires in early pregnancy we aimed to compare the dietary intakes of micronutrients and macronutrients at the first prenatal visit of women who reported continuing to smoke during pregnancy with the intakes of women who were non-smokers.
- **design:** Prospective observational study conducted between June 2014 and March 2016.
- **setting:** Stand-alone tertiary obstetric hospital in an urban setting with approximately 8500 deliveries/ year
- **participants:** Women were recruited at their convenience after sonographic confirmation of an ongoing singleton pregnancy (n=502). Detailed dietary and supplement information was available for 398 women. Women <18 years and those who did not understand English were excluded.
- **primary and secondary outcome measures:** The differences in dietary micronutrients and macronutrients and maternal folate levels between women who continued to smoke in pregnancy compared to non-smokers.
- **results:** Of the 502 women, the mean age was 30.5 (SD 5.6) years, 42.5% were nulliparas, 19.2% were obese and 398 (79.3%) completed the questionnaire satisfactorily. In the 50 (12.6%) current smokers, the micronutrients magnesium, iron, carotene and copper were lower (all p<0.005) whereas sodium and chloride were higher compared with the 348 (87.4%) non-smokers. Smokers reported lower intakes of dietary total folate (p=0.006) compared with non-smokers (i.e. dietary folate equivalents; intake from natural and fortified dietary sources) (p=0.005). Smokers also reported lower intakes of fibre than non-smokers (13.1g (IQR 7.7) vs 16.3g (IQR 8.5), p<0.001). The dietary intakes of former smokers compared favourably with non-smokers.
- **conclusions:** We found that women who continue to smoke during pregnancy have serious dietary inadequacies which potentially may aggravate fetal growth restriction associated with direct toxicity from cigarettes. This provides a further reason to promote smoking cessation interventions in pregnancy, but also highlights the need for dietary and supplementation interventions in women who continue to smoke.
- **Trial registration:** n/a

Word count = 298/300

For peer review only

Strengths and limitations of this study

- This study was conducted prospectively with a well characterised study population.
- The detailed dietary and supplement questionnaires were supervised and analysis of this data included up to date brand level information including details on food fortification with folate.
- Collection of the information in early pregnancy was conducted to minimize the recall bias associated with completion of the questionnaires after delivery.
- Convenience recruitment was used rather than consecutive recruitment.
- The information on smoking in this study was self-reported and not validated by biochemical testing. However, any non-disclosers would be analysed with the non-smokers and it is likely that the dietary differences we found would be even greater if smoking could be verified by biochemical screening.

For peer review only

INTRODUCTION

Maternal smoking is strongly associated with an increase in adverse pregnancy outcomes including miscarriage, preterm delivery, congenital malformations and stillbirth.[1,2] In particular, it is associated with an increased risk of intrauterine fetal growth restriction (IUGR) and the risk increases with the number of cigarettes smoked. Smoking is an important modifiable risk factor for IUGR because cessation of smoking in the first half of pregnancy may normalize fetal growth.[3] Yet, many women continue to smoke throughout pregnancy.

Fetal growth and lifelong pregnancy outcomes are also influenced by maternal nutrition.[4,5] Deficiencies of both maternal micronutrients and macronutrients have been associated with aberrant fetal growth. In a systematic review and meta-analysis of 62 studies from developed countries, pregnant women had dietary folate, iron and vitamin D dietary intakes consistently below nutrient recommendations.[6] Forty-one of the studies, however, did not report maternal smoking status.

This prospective observational study compared the dietary intakes of micro- and macronutrients of self-reported current smokers with the intakes of non-smokers at the first antenatal visit in a large maternity hospital.

METHODS

Women were recruited at their convenience after sonographic confirmation of an ongoing singleton pregnancy during their first prenatal visit between June 2014 and March 2016. Women with multiple pregnancies or women who did not understand English were excluded. Clinical and sociodemographic details were computerized routinely by a trained midwife using a standard questionnaire and a barcode system. The hospital questionnaire included questions on whether the woman had never smoked, had

For peer review only

stopped smoking before her first visit or was continuing to smoke. The number of cigarettes smoked per day was also recorded as 1-5/day, 6-10/day or >10/day. Categorization of Body Mass Index (BMI) was based on measurement of maternal weight and height. Birth outcomes were computerized immediately after delivery. Written informed consent was obtained.

All women were asked to complete a retrospective four day diet history (DH) before they left the clinic. This was combined with a previously validated food frequency questionnaire (FFQ) which had been customized to capture food fortification at brand level in Ireland.[7]

Quantitative food intake data from the DH was divided by four to derive the average daily intake for each food. These data were entered into Nutritics Version 3.7 software to convert the food intakes into nutrient measurements. To overcome the possible limitation associated with nutritional surveys of under-reporting, a calculation was applied to identify any likely under-reporters which is based on energy intake, basal metabolic rate and physical activity level.[8,9] Information on socioeconomic status was analysed using questions from the European Union Survey on Income and Living Conditions (EU SILC).[10] All women had a full blood count taken and other biomarkers including serum folate, whole blood folate and red blood cell (RBC) folate levels were obtained at the same visit.

Data from the questionnaires were anonymized and entered into Microsoft Excel and exported for statistical analysis using SPSS Version 20 (IBM Corporation, Armonk, New York). Continuous data were assessed for normality by visual inspection of the histogram and box-plot and calculation of the kurtosis and skewness. Descriptive statistics were used to describe the characteristics of the study population. The Kruskal Wallis test for non-parametric data was used to assess the difference between nutrient

For peer review only

intakes in women who were current smokers compared to non-smokers. Tests for statistical significance were performed with significance set at the 95% level (i.e. $p < 0.05$). The study was approved by the Hospital's Research Ethics Committee.

RESULTS

Of the 502 women recruited, 398 (79.3%) completed the dietary questionnaire satisfactorily. The characteristics of the study population is shown in Table 1. The study population was similar to the Hospital population.[11] Women cited time constraints as the main reason for not completing the dietary questionnaire and those who did fill out the dietary questionnaire were more likely to be nulliparous (p=0.012) and Irish-born (p<0.001) (Table 1). Comparing current smokers to non-smokers, they were younger (p<0.001) with a higher percentage of relative income poverty compared to the non-smokers (non-smokers; 21.6% (40/185) vs. current smokers; 56.5% (13/23), p=0.009). As expected, the mean birth weight was lower in smokers compared with non-smokers (Table 2).

Table 3 shows the dietary intakes of the overall study group and analyses the intakes according to whether the women reported they were current smokers at the first visit or not. The micronutrients magnesium, iron, carotene, copper, vitamin C and riboflavin were lower in smokers compared with non-smokers but the micronutrients sodium and chloride were higher. Compared with non-smokers, women who smoked had lower levels of dietary folate (p = 0.006, Table 3) and were less likely to take pre-pregnancy folic acid (p<0.001, Table 4). Women who smoked had a lower fibre intake compared with non-smokers (p<0.001) (Table 3).

For peer review only

There was no relationship between the reported number of cigarettes smoked per day (1-5 cigarettes/day compared to ≥ 6 cigarettes/day) and macronutrient and micronutrient intakes. This may have been due to a small sample size.

Comparing the dietary intakes of former smokers (n=155) with never smokers (n=193), the former smokers had superior intakes of the micronutrients iron, zinc, vitamin B12, vitamin B2 (riboflavin) and Vitamin B3 (niacin) (Supplementary Tables 1 and 2). They had higher intakes in protein, sodium and chloride. Due to the similar, if not more favourable intake across all other micronutrients, the non-smokers and former smokers were treated as one group to compare with current smokers.

Table 4 compares the dietary folate intakes for smokers with non-smokers. Non-smokers reported a greater total dietary folate intake (p=0.006) with a superior intake of natural dietary folate compared to smokers (median 193.9 mcg (interquartile range (IQR) 93.9) vs median 174.1 mcg (IQR 174.1) (p=0.029)). However, both groups had a low percentage of women meeting the World Health Organization (WHO) dietary folate equivalent recommended for pregnancy (non-smokers; 6.3% vs smokers; 6.0%) and breastfeeding (non-smokers; 10.9% vs smokers; 6%). This dietary information is supported by the maternal folate measured at the first prenatal visit. Non-smokers had higher serum folate levels (35.2 ± 17.0 nmol/l vs 22.3 ± 19.5nmol/l) and red cell folate levels (1195.1 ± 440.0 nmol/l vs. 820.0 ± 362.5 nmol/l) with 71.3% (248) of the non-smokers achieving the optimal RBC folate level of 906 nmol/l compared with only 38.0% (19) of the smokers.

Of the 348 women in the non-smoking group, 63.8% (222) were plausible energy intake reporters compared to 68% (34) of the women in the current smoker group. Comparing the dietary intakes including any plausible energy intake reporters, lower intakes of fibre (p= 0.004), iron, copper, magnesium, carotene (all p<0.05) and folic acid

For peer review only - http://bmjopen.bmj.com/site/about/guidelines.xhtml

(p=0.009) and a higher chloride intake ($p<0.05$) remained in the current smokers compared with non-smokers (Supplementary Tables 3 and 4).

DISCUSSION

Based on self-reporting at the first prenatal visit, we found that women who continued to smoke during pregnancy were more likely to have deficiencies of both dietary micronutrients and macronutrients than women who did not smoke. Interestingly, women who had stopped smoking had dietary intakes which compared favourably with women who never smoked.

Maternal smoking is strongly associated with adverse clinical outcomes for both the woman and her offspring but our observations raise the possibility that dietary deficiencies may contribute, for example, to the risk of fetal growth restriction. The decrease in total dietary folate in smokers was also associated with a decreased rate of initiation of pre-pregnancy Folic Acid supplementation which increases the risk of a pregnancy complicated by a Neural Tube Defect (NTDs). The findings in former smokers also suggests that cessation of smoking during pregnancy results not only in the fetal avoidance of cigarette toxins but also in improvements in the availability for fetal development of important micronutrients such as iron, zinc, riboflavin, niacin and vitamin B12 ($p<0.05$ for all).

A strength of our study was that it was conducted prospectively and the study population was well characterized. The detailed questionnaires on diet and supplements were supervised and analysis of this data considered up-to-date brand level information, including details on food fortification. The information was collected in early pregnancy to minimize the recall bias associated with the completion of questionnaires after childbirth.

For peer review only - http://bmjopen.bmj.com/site/about/guidelines.xhtml

A limitation of the study was that convenience recruitment was used rather than consecutive recruitment which is not feasible in a busy maternity service with a single interviewer. The information on smoking was self-reported and not validated by biochemical testing. However, any non-disclosures of smoking were analysed with the non-smokers and thus, it is likely that the dietary differences we found would be even greater if smoking could be verified by biochemical screening. It is also difficult to compare our findings on the relationship between dietary intakes and smoking behaviour in our study with previous reports because surveys on dietary intakes in adults usually excluded women who were pregnant or breastfeeding.[12]

Outside of pregnancy, the dietary intakes of smokers compare unfavourably with non-smokers.[13-15] Data from 11,260 adults aged 19-74 years from the second National Health and Nutrition Examination Survey (NHANES) were analyzed to determine food and nutrient differences between smokers and non-smokers.[12] In smokers, there was a lower intake of Vitamins A and C, folate and fibre and the intakes decreased as cigarette consumption increased. Smokers were less likely to consume fruit and vegetables, high fibre grains, low fat milk as well as vitamin and mineral supplements. These findings raised concerns that the lifelong risk of cancer was increased not only by cigarette smoking but also by dietary deficiencies.

Further studies analysed the dietary content of smokers revealing a low intake in sources of important micronutrients. In a random sample of 5,553 men and women between 16 and 74 years of age surveyed in England, low consumers of fruit and vegetables were more likely to be young, male or current smokers than higher consumers.[16] Attitudes about healthy diets were more important than knowledge about a healthy diet.

In a study of women booked for antenatal care in 1982-4, women who smoked heavily (> 15 cigarette per day) (n=72) were compared with controls (n=97).[17] The intakes

For peer review only

of micronutrients were lower in smokers at both 28 and 36 weeks, and smokers reduced their intakes more in late pregnancy. It was estimated that 2.4–7.2% of the reduction in birth weight was explained by decreased nutrient intakes between 28 and 36 weeks.

In a study of 774 pregnant women attending an antenatal clinic in England from 1994-6, a seven day food diary was completed at approximately 17 weeks gestation as part of a structured interview.[18] Women were classified as smokers based on self-reporting or a serum cotinine >14ng/ml. After analysis by multiple linear regression, smokers had lower intakes of most micronutrients, including vitamin C, folate, iron and zinc. The smokers also had lower intakes of protein and fibre. This study, however, was confined to nulliparas with uncomplicated pregnancies.

A study comparing 60 smokers to 80 non-smokers in pregnancy demonstrated the oxidative stress associated with smoking through elevated levels of the oxidative malondialdehyde and corresponding lower levels of known antioxidants such as vitamin E, vitamin A and beta carotene in the plasma of smokers and cord blood of their infants compared to non-smokers and their infants.[19] Smoking produces free radicals that deplete antioxidant levels and these effects could be amplified by poor dietary intake of these important micronutrients.

The reasons for the differences in the dietary intakes between smokers and non-smokers are generally unexplained. One factor that may contribute is the impact of smoking on taste perception. A study conducted with 83 smokers and 48 non-smokers (male and female) using electrogustometry detected taste disturbance in the smokers that was dependent on the intensity of smoking. A follow-up of 24 of the smokers who quit smoking demonstrated a rapid recovery in taste sensitivity within two weeks in certain loci of the tongue and up to 12 months at other sites.[20]

For peer review only

The daily requirement for many micronutrients increases in pregnancy to meet the higher physiologic demands.[21] There are both short and long term consequences of fetal undernutrition. A Norwegian prospective cohort study of 66,000 pregnant women conducted in 2002-8 used a food frequency questionnaire (FFQ) to assess dietary habits and established three patterns: “prudent” (vegetables, fruits, oils, water as beverage, whole grain cereals and fibre rich bread), “western” (salty and sweet snacks, white bread, desserts and processed meats) and “traditional” (potatoes, fish). After adjustment for covariates, higher scores for the “prudent” dietary choices were associated with a decreased risk of preterm delivery (0.88, 95% CI 0.80-0.97). However, there was no independent association with the “western” diet pattern and preterm delivery. It was concluded that low adherence to the “prudent” pattern is a stronger indicator of unhealthy diet than intake of the western diet.[22]

Maternal smoking is associated with higher rates of small for gestational age infants but fetal growth retardation can extend beyond that cut-off point e.g. when an infant born at 3kg fails to reach their growth potential of 3.5kg. Fetal growth is dependent upon their nutrient uptake and the start point of this process is the maternal intake. A study based in India from 1994-7 of 797 pregnant women studied maternal diet and neonatal outcomes. There was a strong correlation between consumption of green leafy vegetables (GLVs), milk and fruit with a higher birth weight. For GLVs, it was shown that women who consumed them on alternate days delivered infants almost 200g heavier than those who never ate them. Furthermore, there were correlations between serum vitamin C and RBC folate levels and birth weight.[5] Considering the long-term consequences of undernutrition, the implications of this on the fetus are described by Barkers hypothesis with an increase in Type 2 diabetes mellitus, hypertension and coronary heart disease.[4,23]

A systematic review and meta-analysis of 13 articles found a pooled odds ratio (OR) of NTDs in offspring of smokers of 1.03 (95% CI =0.80-1.33). When subgroup analysis

For peer review only

was performed based on geographic region, the NTD risk was 1.39 (95% CI = 1.18-1.64) in Europe and 0.88 (95% CI 0.66-1.17) in the US.[24] The transatlantic difference may reflect that the diet of smokers in Europe is less likely to be fortified with folate where fortification is voluntary and not mandatory. Analysis by NTD type also revealed a relationship between maternal smoking and spina bifida (OR 1.55 (95% CI = 1.06-2.26).[24] Therefore, inadequate dietary folate and decreased use of supplementation may contribute to the slight increased risk of NTDs in smokers. The results of our study demonstrated that current smokers were less likely than non-smokers to achieve the optimal RBC folate level of 906nmol/L associated with prevention of NTDs. Above this optimal RBC folate concentration, the risk of neural tube defects is reported to reduce to <8/10,000 live births.[25]

CONCLUSIONS

Our findings that pregnant women who continue to smoke have dietary inadequacies strengthens the public health case for screening women in early pregnancy and for offering support for smoking cessation. The dietary intakes of former smokers suggest that dietary intakes improve after smoking cessation. For those women that continue to smoke in pregnancy, it also highlights the need to advise women about healthy eating behaviours and about the need to start Folic Acid supplementation.

ACKNOWLEDGEMENTS

This work was supported by the all–island body Safefood. We also thank the Friends of the Coombe for their support. We thank the women who participated and our laboratory and midwifery colleagues for their assistance.

For peer review only

AUTHOR CONTRIBUTIONS

EOM contributed to the conception and design of the study, collected data, performed data analysis and wrote and edited the manuscript. SC conducted the primary study involving patient recruitment, issuing questionnaires, analysis of food diaries and gathering data and contributed to the conception and design of this study and drafting of the manuscript. RK and SC were involved in the conception and design of this study, data analysis and contributed to writing and editing. AM was involved in conception and design of the study, drafting sections of the manuscript and approving the final submitted version. MT contributed to the conception and design of the study, analysis of data and writing and editing of the manuscript.

FUNDING

This work was supported by the all island body *Safe*food (grant number 02-2015).

DECLARATION OF INTERESTS

None of the authors have any conflicts of interest to declare.

PATIENT CONSENT

Written consent was obtained.

ETHICAL APPROVAL

The study was approved by the Coombe Women and Infants University Hospital Research and Ethics Committee (REC) – reference number 27-2013.

For peer review only - http://bmjopen.bmj.com/site/about/guidelines.xhtml

DATA SHARING STATEMENT

Extra data is available by emailing eimer.om@gmail.com.

REFERENCES

1. Shea AK, Steiner M. Cigarette smoking during pregnancy. *Nicotine Tob. Res*. 2008;10:267-278
2. Cnattingius S. The epidemiology of smoking during pregnancy: smoking prevalence, maternal characteristics, and pregnancy outcomes. *Nicotine Tob Res*, 2004;6:125-140
3. Blatt K, Moore E, Chen A, et al. Association of Reported Trimester-Specific Smoking Cessation and Fetal Growth Restriction. *Obstet Gynecol* 2015;125:1452-1459.
4. Barker DJ, Winter PD, Osmond C, et al. Weight in infancy and death from ischaemic heart disease. *Lancet* 1989;2:577-580
5. Fall CH, Yajnik CS, Rao S, et al. Micronutrients and fetal growth. *J Nutr* 2003;133:1747-1756
6. Blumfield ML, Hure AJ, Macdonald-Wicks L, et al. Systematic review and meta-analysis of energy and macronutrient intakes in pregnancy in developed countries. *Nutr Rev* 2012;6:322-336
7. McNulty B, Pentieva, K, Marshall, B, et al. Women's compliance with current folic acid recommendations and achievement of optimal vitamin status for preventing neural tube defects. *Hum Reprod*. 2011;26:1530-1536
8. Goldberg GR, Black AE, Jebb SA, et al. Critical evaluation of energy intake data using fundamental principles of energy physiology: 1. Derivation of cut-off limits to identify under-recording. *Eur J Clin Nutr* 1991;45:569-581.

9. Black AE. Critical evaluation of energy intake using the Goldberg cut-off for energy intake: basal metabolic rate. A practical guide to its calculation, use and limitations. *Int J Obes Relat Metab Disord* 2000;24:1119-1130.

10. Central Statistics Office (CSO) (2013) Survey on income and living conditions, Cork, Ireland CSO. Available at: http://pdf.cso.ie/www/pdf/20170908023951_Survey_on_Income_and_Living_Conditions_2013_full.pdf

Accessed October 18, 2017

11. Coombe Women and Infants University Hospital Annual Clinical Report 2014. Available at: http://www.coombe.ie/index.php?nodeId=110. Accessed October 24, 2017

12. Subar AF, Harlan LC, Mattson ME. Food and nutrient intake differences between smokers and non-smokers in the US. *Am J Public Health* 1990;80:1323-9.

13. Dallongeville J, Marecaux N, Fruchart JC, et al. Cigarette smoking is associated with unhealthy patterns of nutrient intake: a meta-analysis. *J Nutr* 1998;128:1450-7.

14. Palaniappan U, Jacobs Starkey L, O'Loughlin J, et al. Fruit and vegetable consumption is lower and saturated fat intake is higher among Canadians reporting smoking. *J Nutr* 2001;131:1952-8.

15. Dyer AR, Elliot E, Stamler J, et al. (INTERMAP Research Group). Dietary intake in male and female smokers, ex-smokers, and never smokers: The INTERMAP Study. J *Hum Hypertens* 2003;17:641-54

16. Thompson RL, Margetts BM, Speller VM, et al. The Health Education Authority's health and fruit lifestyle survey 1993: who are the low fruit and vegetable consumers?

J Epidemiol Community Health 1999;53:294-9.

For peer review only

17. Haste FM, Brooke OG, Anderson HR, et al. The effect of nutritional intake on outcome of pregnancy in smokers and non-smokers. *Br J Nutr* 1991;65:347-354

18. Mathews F, Yudkin P, Smith RF, et al. Nutrient intakes during pregnancy: the influence of smoking status and age. *J Epidemiol Community Health* 2000;54:17-23.

19. .Chelchowska M, Ambroszkiewicz J, Gajewska J, Laskowska-Kilta T and Leibschang J. The effect of tobacco smoking during pregnancy on plasma oxidant and antioxidant status in mother and newborn. *Eur J Obstet Gynecol Reprod Biol* 2011;155;132-136

20. Chéruel F, Jarleir M, Sancho-Garnier H. Effect of cigarette smoking on gustatory sensitivity, evaluation of the deficit and of recovery time course after smoking cessation*. Tob Induc Dis* 2017;15:15

21. King JC. Physiology of pregnancy and nutrient metabolism. *Am J Clin Nut* 2000;71:1218-1225.

22. Englund-Ogge L, Brantsaeter AL, Verena LS, et al. Maternal dietary patterns and preterm delivery: results from a large prospective cohort study. *BMJ* 2014;348:1446

23. Micronutrient needs during pregnancy and lactation. Drake VJ et al, updated 2016. Oregon state university. Report online. Available at: http://lpi.oregonstate.edu/mic/life-stages/pregnancy-lactation . Accessed on October 29th 2017

24. Wang M, Wang ZP, Gong R, et al. Maternal smoking during pregnancy and neural tube defects in offspring: a meta-analysis. *Childs Nerv Syst* 2013;30:83-89

25. Daly LE, Kireke PN, Molloy A, et al. Folate levels and neural tube defects-implications for prevention *JAMA* 1995;274:1698-1702

For peer review only - http://bmjopen.bmj.com/site/about/guidelines.xhtml

Table 1. Characteristics of the study population including delivery outcomes

	Study population (n=502)[a]	Women with dietary data (n=398)	Women without dietary data (n=104)	P
Age (years) (mean ± SD)	30.7 ± 5.5	30.5 ± 5.6	31.2 ± 5.0	NS
BMI (kg/m^2) (mean ± SD)	26.0 ± 5.5	25.9 ± 5.4	26.1 ± 5.8	NS
Obese (%)	19.3% (97)	19.2% (76)	20.1%(21)	NS
Nulliparous (%)	42.6% (214)	45.5% (181)	31.7% (33)	0.012
Irish-born (%)	70.1% (352)	76.9% (306)	44.2% (46)	<0.001
Alcohol intake during pregnancy (%)	4.0% (20)	4.8 % (19)	0.9% (1)	NS
Taking FA pre-pregnancy	42.8% (215)	43.2% (172)	41.3% (43)	NS
GA at delivery (weeks) (mean ± SD)	39.5 +/-1.9	39.5 +/- 2.0	39.5 +/- 1.6	NS
Preterm delivery (%)	4.0% (19)	3.7% (14)	5.3% (5)	NS
Birth weight (grams) (mean +/- SD)	3422 ± 573.3	3426.9 ±586.1	3402.5 ±520.9	NS

For peer review only

SGA[b] (%, (n))	8.9% (42)	9.3% (34)	8.5% (8)	NS
Birth weight >4.5kg (%)	1.5% (7)	1.9% (7)	0.0% (0)	NS
Caesarean delivery (%)	28.4% (134)	27.3% (103)	32.9% (31)	NS

[a] Of the 502 women studied, delivery outcomes were available on 472; 378 with dietary data and 94 without dietary data. The remaining women delivered elsewhere.

[b]SGA (Small for gestational age) was calculated as a birth weight ≤10%ile for all deliveries at term (n=364), with gestations grouped week by week e.g. 37.0-37.9 weeks, 38.0-38.9 weeks etc.

NS – not significant

FA – Folic acid

SD – Standard Deviation

BMI – Body Mass Index

GA = gestational age

kg - Kilograms

For peer review only

Table 2. Comparison of characteristics between smokers and non-smokers with dietary data available (n=398)

	All women (n=398)[a]	Smokers (n=50)	Non-smokers (n=348)	p
Age (years) (mean ± SD)	30.5 ± 5.6	27.0 ± 6.44	31.0 ± 5.3	<0.001
BMI (kg/m^2) (mean ± SD)	25.9 ±5.4	25.3 ± 4.7	26.0 ±5.5	0.418
Obese (%)	19.2% (76)	22% (11)	18.7% (65)	0.323
Nulliparous (%)	45.5% (181)	46.0% (23)	45.4% (158)	0.937
Irish-born (%)	76.9% (306)	82% (41)	76.1% (265)	0.739
Alcohol intake during pregnancy (%)	4.8 % (19)	6.1% (3)	4.7% (16)	0.863
GA at delivery (weeks) (mean +/- SD)	39.5+/-2.0	38.9 +/- 2.8	39.6 +/- 1.9	0.040
Preterm delivery(%, n)	3.7% (14)	8.3% (4)	3.0% (10)	0.069
Birth weight (grams)	3426.9 ±	3134.4 ± 622.1	3469.46 ± 569.2	<0.001

For peer review only

(mean ± SD)	586.1			
SGA[b] (%, n)	9.3% (34)	14.6% (7)	8.2%(27)	0.110
Birth weight >4.5kg (%)	1.9% (7)	0.0%	2.1% (7)	0.308
Caesarean delivery (%)	27.3% (103)	16.7% (8)	28.8% (95)	0.055

[a] Of the 398 women studied, delivery outcomes were available on 378; 330 who were non-smokers and 48 who were current smokers. The remaining women delivered elsewhere.

[b]SGA - (Small for gestational age) was calculated as a birth weight </=10%ile for all deliveries at term (n=364), with gestations grouped week by week e.g. 37.0-37.9 weeks, 38.0-38.9 weeks etc.

FA – folic acid

SD – standard deviation

BMI – Body Mass Index

GA = gestational age

For peer review only

Table 3. Daily macronutrient and micronutrient intakes in smokers compared to non-smokers

	Smokers (n=50) (median, IQ range)	Non-smokers (n=348) (median, IQ range)	p
Protein (g)	69.1 (31.3)	74.4 (28.0)	0.168
Carbohydrate (g)	204.3 (95.7)	198.8 (73.7)	0.820
Fat (g)	75.5 (42.8)	72.7 (39.1)	0.421
Saturates (g)	30.3 (15.1)	27.2 (15.7)	0.353
Monounsaturated fat (g)	25.3 (13.7)	24.5 (13.6)	0.928
Polyunsaturated fat (g)	8.8 (7.0)	10.3 (7.0)	0.176
Fibre (g)	13.1 (7.7)	16.3 (8.5)	<0.001
Sodium (mg)	2100.7 (1158.7)	1877.0 (971.9)	0.095
Potassium (mg)	2371.2 (1085.7)	2533.1 (1038.5)	0.305
Calcium (mg)	797.8 (538.06)	788.4 (536.4)	0.601
Magnesium (mg)	196.0 (75.8)	226.9 (100.5)	0.003
Phosphorous (mg)	1060.5 (381.7)	1180.8 (490.1)	0.063
Iron (mg)	7.9 (3.9)	9.5 (4.25)	0.002
Copper (mg)	0.7 (0.4)	0.9 (0.5)	0.001
Zinc (mg)	7.1 (3.9)	7.8 (3.7)	0.227
Chloride (mg)	3187.3 (1678.0)	2843.7 (1345.8)	0.067
Iodine (µg)	91.5 (89.5)	104.5 (77.0)	0.500
Retinol (µg)	312.6 (225.0)	282.9 (242.3)	0.916

For peer review only

Carotene (µg)	1488.3 (3427.4)	3213.2 (4479.5)	0.004
Vitamin D (µg)	2.3 (2.5)	2.4 (2.6)	0.547
Vitamin C (mg)	71.6 (74.3)	83.8 (83.0)	0.035
Vitamin E (µg)	7.1 (5.1)	8.2 (5.6)	0.196
Thiamine (mg)	1.3 (0.6)	1.4 (0.7)	0.100
Riboflavin (mg)	1.3 (0.9)	1.4 (0.7)	0.040
Niacin (mg)	30.5 (16.8)	34.1 (14.4)	0.089
Vitamin B6 (mg)	1.7 (1.0)	1.9 (0.9)	0.451
Vitamin B12 (µg)	4.0 (3.5)	4.0 (2.6)	0.767
Folate (natural and fortified) (µg)	202.1 (127.3)	241.9 (142.8)	0.006

SD – standard deviation

IQ range – Interquartile range

g - grams

mg – milligrams

µg = micrograms

For peer review only

Table 4. Total median (IQR) maternal dietary folate (including fortified folic acid) per day in early pregnancy and hematological measures of folate in non-smokers compared to current smokers (n=398).

	Non-smokers (n=348)	Smokers (n=50)	p
Natural dietary folate intake (mcg)	193.9 (93.9)	174.1 (112.7)	0.029
Fortified dietary folate intake (mcg)	33.8 (50.7)	16.9 (59.4)	0.076
Total dietary folate intake (mcg)	241.9 (142.8)	202.1 (127.1)	0.006
Dietary folate equivalent (mcg)	268.2 (190.7)	214.6 (146.9)	0.005
Achieving WHO DFE for pregnancy (>/= 600 mcg DFE) (%, n)	6.3 % (22)	6.0 % (3)	0.930
Achieving WHO DFE for lactation (>/= 500 mcg DFE) (%,n)	10.9 % (38)	6.0 % (3)	0.285
Pre-pregnancy FA use (%,n)	48.0 % (167)	10.0 % (5)	0.015
Serum folate (nmol/L), (median, IQR)	35.2 +/- 17.0	22.3 +/- 19.5	<0.001

For peer review only

Red blood cell (RBC) folate (nmol/L), (mean, SD)	1195.1 +/- 440.0	820.0 +/- 362.5	<0.001
Achieving optimal RBC folate (%,n)[a]	71.3 % (248)	38.0 % (19)	<0.001

IQR– interquartile range

mcg – micrograms

WHO – World Health Organisation

SD – standard deviation

DFE – Dietary folate equivalent. For natural folate from food – 1mcg folate from food is equal to 1mcg DFE. For food fortified with folate 1mcg of folate = 1.7 DFE, as natural folate from food is 50% bioavailable compared to 85% bioavailability from fortified folate and folate supplementation (85/50 = 1.7)

[a] The reported optimal threshold concentration for red blood cell folate is reported as 906nmol/L by Daly et al.[25] At and above this concentration, the risk of neural tube defects (NTDs) was reduced to <8 NTDs/10,000 live births.

For peer review only

Supplementary Table 1:

Comparison of macronutrient intake between never smokers and former smokers

	Never smokers (n=193) (median (IQ range))	Former smokers (n=155) (median (IQ range))	P
Carbohydrate (g)	199.0 (68.2)	197.0 (89.6)	0.535
Protein (g)	72.8 (26.0)	77.0 (29.1)	0.027
Fat (g)	68.8 (40.1)	77.8 (36.4)	0.071
Saturated fat (g)	25.6 (14.2)	28.5 (16.3)	0.211
Monounsaturated fat (g)	24.0 (14.2)	25.5 (12.8)	0.256
Polyunsaturated fat (g)	10.1(7.2)	10.7 (7.3)	0.179
Fibre (g)	16.3 (8.8)	16.2 (8.0)	0.574

IQ range – interquartile range

g – grams

For peer review only

Supplementary Table 2:

Comparison of micronutrient intake between never smokers and former smokers

	Never smokers (n=193) (median (IQ range))	Former smokers (n=155) (median (IQ range))	P
Sodium (mg)	1805.2 (928.7)	2069.6 (988.1)	0.008
Potassium (mg)	2472.9 (946.4)	2631.2 (1127.0)	0.102
Chloride (mg)	2688.6 (1294.4)	3067.6 (1329.7)	0.045
Calcium (mg)	749.0 (505.8)	838.0 (533.0)	0.120
Phosphorous (mg)	1124.5 (461.6)	1212.2 (463.5)	0.055
Iron (mg)	9.1 (3.5)	10.0 (5.5)	0.014
Magnesium (mg)	225.1 (98.0)	229.8 (110.3)	0.334
Zinc (mg)	7.7 (3.8)	8.1 (3.9)	0.041
Copper (mg)	0.9 (0.5)	0.9 (0.5)	0.501
Iodine (mcg)	101.8 (69.8)	106.9 (86.7)	0.080
Retinol (mcg)	274.6 (248.2)	310.9 (231.8)	0.089
Carotene (mcg)	3475.6 (5193.8)	2965.4 (4199.2)	0.300
Vitamin C (mg)	79.9 (88.5)	89.5 (78.2)	0.174
Vitamin D (mcg)	2.2 (2.5)	2.5 (2.9)	0.438
Vitamin E (mcg)	8.0 (5.5)	8.4 (5.5)	0.123

For peer review only

Thiamine B1 (mg)	1.4 (0.7)	1.4 (0.6)	0.293
Riboflavin B2 (mg)	1.4 (0.7)	1.6 (0.8)	0.008
Niacin B3 (mg)	33.3 (14.1)	35.8 (15.0)	0.038
Vitamin B6 (mg)	1.9 (0.8)	1.9 (0.8)	0.191
Vitamin B12 (mg)	3.8 (2.8)	4.1 (2.6)	0.018
Folic Acid (mcg)	234.1 (135.9)	249.0 (148.9)	0.154

IQ – interquartile range

mg – milligrams

mcg - micrograms

For peer review only

Supplementary Table 3: Comparison of macronutrient intakes between smokers and non-smokers (excluding likely energy under-reporters i.e. plausible reporters only)

	Non-smokers (n=222) (median (IQ range))	Current smokers (n=34) (median (IQ range))	P
Carbohydrate (g)	217.3 (72.8)	230.0 (94.2)	0.229
Protein (g)	80.2 (25.1)	78.3 (33.5)	0.566
Fat (g)	84.3 (33.0)	90.6 (34.8)	0.416
Fibre (g)	18.3 (8.1)	14.6 (6.3)	0.004
Saturated fat (g)	31.5 (16.6)	35.5 (13.4)	0.472
Monounsaturated fat (g)	29.0 (12.5)	27.9 (12.8)	0.978
Polyunsaturated fat (g)	12.1 (7.3)	10.8 (8.3)	0.236

IQ range – interquartile range

g - grams

For peer review only

Supplementary Table 4: Comparison of micronutrient intakes between smokers and non-smokers (excluding likely energy under-reporters i.e. plausible reporters only)

	Non-smokers (n=222) (median (IQ range))	Current smokers (n=34) (median (IQ range))	P
Sodium (mg)	2126.2 (991.6)	2499.4 (1092.7)	0.079
Potassium (mg)	2785.8 (965.7)	2764.8 (1037.3)	0.631
Chloride (mg)	3156.8 (1399.0)	3624.4 (1747.9)	0.031
Calcium (mg)	961.9 (589.9)	848.8 (393.2)	0.396
Phosphorous (mg)	1299.2 (451.4)	1137.7 (479.4)	0.113
Iron (mg)	10.4 (4.3)	9.7 (3.7)	0.023
Magnesium (mg)	252.4 (101.0)	214.7 (80.8)	0.012
Zinc (mg)	8.6 (3.4)	8.1 (3.8)	0.357
Copper (mg)	1.0 (0.5)	0.8 (0.3)	0.009
Iodine (mcg)	120.5 (84.9)	124.8 (92.1)	0.889
Retinol (mcg)	344.9 (282.7)	344.2 (248.9)	0.836
Carotene (mcg)	3213.2 (4762.3)	1488.3 (3572.3)	0.016
Vitamin C (mg)	97.0 (90.8)	77.7 (69.6)	0.097
Vitamin D (mcg)	2.6 (2.9)	2.7 (2.5)	0.726
Vitamin E (mcg)	9.2 (5.4)	8.6 (7.2)	0.321

For peer review only

Thiamine B1 (mg)	1.5 (0.6)	1.5 (0.6)	0.486
Riboflavin B2 (mg)	1.7 (0.8)	1.5 (1.0)	0.244
Niacin B3 (mg)	36.1 (13.7)	33.4 (15.7)	0.562
Vitamin B6 (mg)	2.0 (0.8)	2.1 (0.8)	0.645
Vitamin B12 (mg)	4.5 (2.7)	5.1 (3.9)	0.528
Folic Acid (mcg)	266.2 (123.8)	213.4 (135.9)	0.009

IQ range – interquartile range

mg – milligrams

mcg – micrograms

For peer review only

Comparison at the first prenatal visit of the maternal dietary intakes of smokers with non-smokers in a large maternity hospital: a cross sectional study.

Journal:	*BMJ Open*
Manuscript ID	bmjopen-2018-021721.R1
Article Type:	Research
Date Submitted by the Author:	28-Feb-2018
Complete List of Authors:	O'Malley, Eimer<br>Cawley, Shona; University College Dublin, UCD Centre for Human Reproduction; Dublin Institute of Technology<br>Reynolds, Ciara; University College Dublin, UCD Centre for Human Reproduction<br>Kennedy, Rachel; University College Dublin, UCD Centre for Human Reproduction; Dublin Institute of Technology<br>Molloy, Anne; University of Dublin Trinity College, School of Medicine<br>Turner, Michael; University College Dublin
**Primary Subject Heading**:	Obstetrics and gynaecology
Secondary Subject Heading:	Nutrition and metabolism
Keywords:	Fetal medicine < OBSTETRICS, Maternal medicine < OBSTETRICS, PUBLIC HEALTH

SCHOLARONE™ Manuscripts

For peer review only - http://bmjopen.bmj.com/site/about/guidelines.xhtml

Title: Comparison at the first prenatal visit of the maternal dietary intakes of smokers with non-smokers in a large maternity hospital: a cross sectional study

Authors: O'Malley EG[1], Cawley S[1,2], Reynolds CME[1], Kennedy RAK[1,2], Molloy A[3], Turner MJ[1].

Affiliations [1]UCD Centre for Human Reproduction, Coombe Women and Infants University Hospital, Cork St, Dublin 8, Ireland

[2] School of Biological Sciences, Dublin Institute of Technology, Kevin St., Dublin 8, Ireland

[3] School of Medicine, Trinity College Dublin, Dublin 2, Ireland.

Corresponding Author: E O'Malley

Email: eimer.om@gmail.com

Telephone: +35314085571

Key words: Maternal dietary intakes, Maternal smoking, Intrauterine fetal growth, Small for gestational age

Word Count: 3082 words

For peer review only

ABSTRACT

- **Objectives:** Using detailed dietary and supplement questionnaires in early pregnancy we aimed to compare the dietary intakes of micronutrients and macronutrients at the first prenatal visit of women who reported continuing to smoke during pregnancy with the intakes of women who were non-smokers.
- **Design:** Cross sectional study conducted between June 2014 and March 2016.
- **Setting:** Stand-alone tertiary maternity hospital in an urban setting with approximately 8000 deliveries/ year
- **Participants:** Women were recruited at their convenience after sonographic confirmation of an ongoing singleton pregnancy (n=502). Detailed dietary and supplement information was available for 398 women. Women <18 years and those who did speak English fluently were excluded.
- **Primary and secondary outcome measures:** The differences in dietary micronutrients and macronutrients and maternal folate levels between women who continued to smoke in pregnancy compared to non-smokers.
- **Results:** Of the 502 women, the mean age was 30.5 (SD 5.6) years, 42.5% were nulliparas, 19.2% were obese and 398 (79.3%) completed the questionnaire satisfactorily. In the 50 (12.6%) current smokers, the micronutrients magnesium, iron, carotene and copper were lower (all p<0.005) whereas sodium and chloride were higher compared with the 348 (87.4%) non-smokers. Smokers reported lower intakes of dietary total folate (p=0.006) compared with non-smokers (i.e. dietary folate equivalents; intake from natural and fortified dietary sources) (p=0.005). Smokers also reported lower intakes of fibre than non-smokers (13.1g (IQR 7.7) vs 16.3g (IQR 8.5), p<0.001). The dietary intakes of former smokers compared favourably with non-smokers.

For peer review only

- **Conclusions:** We found that women who continue to smoke during pregnancy have serious dietary inadequacies which potentially may aggravate fetal growth restriction associated with direct toxicity from cigarettes. This provides a further reason to promote smoking cessation interventions in pregnancy, but also highlights the need for dietary and supplementation interventions in women who continue to smoke.
- **Trial registration:** n/a

Word count = 298/300

For peer review only

Strengths and limitations of this study

- This study was a cross-sectional study with a well characterised study sample.
- The detailed dietary and supplement questionnaires were supervised and analysis of this data included up to date brand level information including details on food fortification with folate.
- Collection of the information in early pregnancy was conducted to minimise the recall bias associated with completion of the questionnaires after delivery.
- A limitation of the study is that convenience recruitment was used rather than consecutive recruitment, as the later was not possible with a single interviewer.
- The information on smoking in this study was self-reported and not validated by biochemical testing. However, any non-disclosers would be analysed with the non-smokers and it is likely that the dietary differences we found would be even greater if smoking could be verified by biochemical screening.

For peer review only

INTRODUCTION

Maternal smoking is strongly associated with an increase in adverse pregnancy outcomes including miscarriage, preterm delivery, congenital malformations and stillbirth. [1,2] In particular, it is associated with an increased risk of intrauterine fetal growth restriction (IUGR) and the risk increases with the number of cigarettes smoked. Smoking is an important modifiable risk factor for IUGR because cessation of smoking in the first half of pregnancy may normalize fetal growth. [3] Yet, many women continue to smoke throughout pregnancy.

Fetal growth and lifelong pregnancy outcomes are also influenced by maternal nutrition. [4,5] Deficiencies of both maternal micronutrients and macronutrients have been associated with aberrant fetal growth. In a systematic review and meta-analysis of 62 studies from developed countries, pregnant women had dietary folate, iron and vitamin D dietary intakes consistently below nutrient recommendations. [6] Forty-one of the studies, however, did not report maternal smoking status which may influence dietary intakes.

This cross-sectional study compared the dietary intakes of micro- and macronutrients of self-reported current smokers with the intakes of non-smokers at the first antenatal visit in a large maternity hospital.

For peer review only

METHODS

Women were recruited at their convenience after sonographic confirmation of an ongoing singleton pregnancy during their first prenatal visit between June 2014 and March 2016. Women with multiple pregnancies or women who did not speak English fluently were excluded. Clinical and sociodemographic details were computerized routinely by a trained midwife using a standard questionnaire and a barcode system. The

hospital questionnaire included questions on whether the woman had never smoked, had stopped smoking before her first visit or was continuing to smoke. The number of cigarettes smoked per day was also recorded as 1-5/day, 6-10/day or >10/day. The maternal weight and height were measured by a midwife at the first antenatal visit and the Body Mass Index (BMI) was calculated and categorised. This is more accurate than a self-reported pre-pregnancy weight. [7] Body weight, BMI and body composition have been shown to not vary significantly across the first trimester in a study of 1000 women.[8] Written informed consent was obtained.

All women were asked to complete a retrospective four-day diet history (DH) at the first antenatal visit. This was combined with a previously validated food frequency questionnaire (FFQ) which had been customized to capture food fortification with folate at brand level in Ireland.[9]

Quantitative food intake data from the DH was divided by four to derive the average daily intake for each food. These data were entered into Nutritics Version 3.7 software to convert the food intakes into nutrient measurements. The nutrient requirement reference levels for pregnancy (either average requirement, population reference intake or adequate intake) were obtained from the European Food Safety Authority (EFSA) guideline [10]. Levels for Sodium, Chloride and Vitamin E are not established.

To overcome the possible limitation associated with nutritional surveys of under-reporting, a calculation was applied to identify any likely under-reporters which is based on energy intake, basal metabolic rate and physical activity level.[11,12] Based on the reported physical activity level (PAL), the lowest plausible PAL threshold (from 1.45-2.20) was determined. If the energy intake (EI) to basal metabolic rate (BMR) ratio fell

For peer review only

below the plausible threshold for their PAL then they were identified as a dietary under-reporter. Plausible energy intake reporters could be identified in this way.

Preliminary analyses conducted demonstrated that dietary composition was similar between never smokers and former smokers (Supplementary tables 1 and 2), therefore, analyses combined never and former smokers into one “non-smokers” group.

-Information on socioeconomic status was analysed using questions from the European Union Survey on Income and Living Conditions (EU SILC). [13] All women had a full blood count taken and other biomarkers including serum folate, whole blood folate and red blood cell (RBC) folate levels were obtained at the same visit. Information on birth outcomes were obtained from the hospital computerized database which is updated immediately after delivery.

Data from the questionnaires were anonymised and entered into Microsoft Excel and exported for statistical analysis using SPSS Version 20 (IBM Corporation, Armonk, New York). Continuous data were assessed for normality by visual inspection of the histogram and box-plot and calculation of the kurtosis and skewness. Descriptive statistics were used to describe the characteristics of the study sample. The Kruskal-Wallis test for non-parametric data was used to assess the difference between nutrient intakes in women who were current smokers compared to non-smokers. Tests for statistical significance were performed with the level for significance set at $p<0.05$. The study was approved by the Hospital's Research Ethics Committee.

Patient and Public Involvement

The authors were aware of the increased risk of adverse pregnancy outcomes associated with maternal smoking and also the association of smoking with poor dietary quality compared to non-smokers. This was the stimulus for our research question.

For peer review only

The patients were not involved in the study design or the recruitment process however the Hospital Research Ethics Committee includes members of the public involved in reviewing the methodology, patient information leaflets and questionnaires.

The results of our study were not directly disseminated to the study population. They will be presented locally to educate staff on our findings with the aim of changing practice to benefit our patients.

RESULTS

Of the 502 women recruited, 398 (79.3%) completed the dietary questionnaire satisfactorily. The characteristics of the study sample is shown in Table 1. The study sample was similar to the Hospital population. [14] Women cited time constraints as the main reason for not completing the dietary questionnaire and those who did fill out the dietary questionnaire were more likely to be nulliparous (p=0.012) and Irish-born (p<0.001) (Table 1). Comparing current smokers to non-smokers, they were younger (p<0.001) with a higher percentage of relative income poverty compared to the non-smokers (non-smokers; 21.6% (40/185) vs. current smokers; 56.5% (13/23), p=0.009). As expected, the mean birth weight was lower in smokers compared with non-smokers (Table 2).

Table 3 presents the dietary intakes overall and stratified by smoking status. The micronutrients magnesium, iron, carotene, copper, vitamin C and riboflavin were lower in smokers compared with non-smokers but the micronutrients sodium and chloride were higher. Compared with non-smokers, women who smoked had lower levels of dietary folate (p = 0.006, Table 3) and were less likely to take pre-pregnancy folic acid (p<0.001, Table 4). Women who smoked had a lower fibre intake compared with non-smokers (p<0.001) (Table 3).

For peer review only

There was no relationship between the reported number of cigarettes smoked per day (1-5 cigarettes/day compared to ≥ 6 cigarettes/day) and macronutrient and micronutrient intakes.

Comparing the dietary intakes of former smokers (n=155) with never smokers (n=193), the former smokers had superior intakes of the micronutrients iron, zinc, vitamin B12, vitamin B2 (riboflavin) and Vitamin B3 (niacin) (Supplementary Tables 1 and 2). They had higher intakes in protein, sodium and chloride.

Table 4 compares the dietary folate intakes for smokers with non-smokers. Non-smokers reported a greater total dietary folate intake (p=0.006) with a superior intake of natural dietary folate compared to smokers (median 193.9 mcg (interquartile range (IQR) 93.9) vs median 174.1 mcg (IQR 174.1) (p=0.029)). However, both groups had a low percentage of women meeting the World Health Organization (WHO) dietary folate equivalent recommended for pregnancy (non-smokers; 6.3% vs smokers; 6.0%) and breastfeeding (non-smokers; 10.9% vs smokers; 6%). This dietary information is supported by the maternal folate measured at the first prenatal visit. Non-smokers had higher serum folate levels (35.2 ± 17.0 nmol/L vs 22.3 ± 19.5nmol/L) and red cell folate levels (1195.1 ± 440.0 nmol/L vs. 820.0 ± 362.5 nmol/L) with 71.3% (248) of the non-smokers achieving the optimal RBC folate level of 906 nmol/L compared with only 38.0% (19) of the smokers.

Of the 348 women in the non-smoking group, 63.8% (222) were plausible energy intake reporters compared to 68% (34) of the women in the current smoker group. Comparing the dietary intakes including any plausible energy intake reporters, lower intakes of fibre (p= 0.004), iron, copper, magnesium, carotene (all p<0.05) and folic acid (p=0.009) and a higher chloride intake (p<0.05) remained in the current smokers compared with non-smokers (Supplementary Tables 3 and 4).

For peer review only

DISCUSSION

Based on self-reporting at the first prenatal visit, we found that women who continued to smoke during pregnancy were more likely to have deficiencies of both dietary micronutrients and macronutrients than women who did not smoke. Interestingly, women who had stopped smoking had dietary intakes which compared favourably with women who never smoked.

Maternal smoking is strongly associated with adverse clinical outcomes for both the woman and her offspring but our observations raise the possibility that dietary deficiencies may contribute, for example, to the risk of fetal growth restriction. The decrease in total dietary folate in smokers was also associated with a decreased rate of initiation of pre-pregnancy Folic Acid supplementation which increases the risk of a pregnancy complicated by a Neural Tube Defect (NTDs). The findings in former smokers also suggests that cessation of smoking during pregnancy results not only in the fetal avoidance of cigarette toxins but also in likely improvements in the maternal diet with increasing intakes of important micronutrients such as iron, zinc, riboflavin, niacin and vitamin B12 ($p<0.05$ for all).

Our findings that smokers have a poor dietary composition relative to non-smokers is consistent with many previous studies, including those among the general adult population [15-18] and pregnant women [19,20].

Data from 11,260 adults aged 19-74 years from the second National Health and Nutrition Examination Survey (NHANES) were analyzed to determine food and nutrient differences between smokers and non-smokers. [21] In smokers, there was a lower intake of

For peer review only - http://bmjopen.bmj.com/site/about/guidelines.xhtml

Vitamins A and C, folate and fibre and the intakes decreased as cigarette consumption increased. Smokers were less likely to consume fruit and vegetables, high fibre grains, low fat milk as well as vitamin and mineral supplements. These findings raised concerns that the lifelong risk of cancer was increased not only by cigarette smoking but also by dietary deficiencies.

A Swiss population study of 2301 men and 2306 women reported that current heavy smokers consumed less total vegetable proteins, carbohydrates, fibre, betacarotene, fruit and vegetables compared to non-smokers (p values all significant). [22] They also drank more alcohol and coffee ($p<0.0001$ and $p<0.005$ respectively). The female smokers also consumed less complex carbohydrates ($p<0.002$) and less iron ($p<0.02$). As in this study, the diet of ex-smokers was similar to never smokers. This demonstrates that it is likely that quitting smoking is associated with other positive lifestyle modifications including improvement of diet.

A study comparing 60 smokers to 80 non-smokers in pregnancy demonstrated the oxidative stress associated with smoking through elevated levels of the oxidative malondialdehyde and corresponding lower levels of known antioxidants such as vitamin E, vitamin A and beta carotene in the plasma of smokers and cord blood of their infants compared to non-smokers and their infants.[23] Smoking produces free radicals that deplete antioxidant levels and these effects could be amplified by poor dietary intake of these important micronutrients.

The reasons for the differences in the dietary intakes between smokers and non-smokers are generally unexplained. One factor that may contribute is the impact of smoking on taste perception. A study conducted with 83 smokers and 48 non-smokers (male and female) using electrogustometry detected taste disturbance in the smokers that was dependent on the intensity of smoking. A follow-up of 24 of the smokers who quit smoking

demonstrated a rapid recovery in taste sensitivity within two weeks in certain loci of the tongue and up to 12 months at other sites.[24]

The daily requirement for many micronutrients increases in pregnancy to meet the higher physiologic demands. [25] There are both short and long term consequences of fetal undernutrition. A Norwegian prospective cohort study of 66,000 pregnant women conducted in 2002-8 used a food frequency questionnaire (FFQ) to assess dietary habits and established three patterns: "prudent" (vegetables, fruits, oils, water as beverage, whole grain cereals and fibre rich bread), "western" (salty and sweet snacks, white bread, desserts and processed meats) and "traditional" (potatoes, fish). After adjustment for covariates, higher scores for the "prudent" dietary choices were associated with a decreased risk of preterm delivery (0.88, 95% CI 0.80-0.97). However, there was no independent association with the "western" diet pattern and preterm delivery. It was concluded that low adherence to the "prudent" pattern is a stronger indicator of unhealthy diet than intake of the western diet. [26]

Maternal smoking is associated with higher rates of small for gestational age infants but fetal growth retardation can extend beyond that cut-off point e.g. when an infant born at 3kg fails to reach their growth potential of 3.5kg. Fetal growth is dependent upon their nutrient uptake and the start point of this process is the maternal intake. A study based in India from 1994-7 of 797 pregnant women studied maternal diet and neonatal outcomes. There was a strong correlation between consumption of green leafy vegetables (GLVs), milk and fruit with a higher birth weight. For GLVs, it was shown that women who consumed them on alternate days delivered infants almost 200g heavier than those who never ate them. Furthermore, there were correlations between serum vitamin C and RBC folate levels and birth weight. [5] Considering the long-term consequences of undernutrition, the implications of this on the fetus are described by Barkers hypothesis with an increase in Type 2 diabetes mellitus, hypertension and coronary heart disease.[4,27]

For peer review only

A Canadian study conducted from 2001-7 of 1,646 self-reported smokers compared to 19,292 non-smokers, reported an increase in adverse perinatal and neonatal outcomes amongst smokers. There was an increased risk of preterm delivery (22.2% vs 12.4%, $p<0.05$), intrauterine fetal demise (OR 2.4 (95% CI 1.4-4.2), a mean birth weight approximately 200 grams lower in the current smokers ($p<0.05$) and an increased risk of neonatal death (1.2% vs 0.6, $p<0.05$). [28]

A systematic review and meta-analysis of 13 articles found a pooled odds ratio (OR) of NTDs in offspring of smokers of 1.03 (95% CI =0.80-1.33). When subgroup analysis was performed based on geographic region, the NTD risk was 1.39 (95% CI = 1.18-1.64) in Europe and 0.88 (95% CI 0.66-1.17) in the US. [29] The transatlantic difference may reflect that the diet of smokers in Europe is less likely to be fortified with folate where fortification is voluntary and not mandatory. Analysis by NTD type also revealed a relationship between maternal smoking and spina bifida (OR 1.55 (95% CI = 1.06-2.26). [24] Therefore, inadequate dietary folate and decreased use of supplementation may contribute to the slight increased risk of NTDs in smokers. The results of our study demonstrated that current smokers were less likely than non-smokers to achieve the optimal RBC folate level of 906nmol/L associated with prevention of NTDs. Above this optimal RBC folate concentration, the risk of neural tube defects is reported to reduce to <8/10,000 live births. [30]

Women who continue to smoke in pregnancy may benefit from dietary intervention. A study of 862 women and infant pairs assessed maternal dietary quality at 24-28 weeks gestation. Lower dietary quality was associated with lower educational attainment, maternal smoking, obesity (pre-pregnancy) and a lack of exercise during pregnancy. It was found that increased dietary quality was associated with a reduced likelihood of a small for gestational

For peer review only

age infant. Furthermore, a higher diet quality was positively associated with birth weight amongst former and current smokers. [31]

A study of two mother and infant cohorts (2461 pairs in Spain and 889 pairs in Greece) found that a high Mediterranean diet (MD - vegetables, legumes, fruit, nuts, cereals, seafood and fish and dairy products) adherence was associated with a lower risk of a growth restricted infant (risk ratio 0.5, 95% CI 0.3-0.9). When the analysis was stratified according to smoking status, higher MD adherence was associated with higher birth weight and birth length in the infants of mothers who continued to smoke. [32]

A strength of our study was that it was conducted prospectively and the study sample was well characterized. The detailed questionnaires on diet and supplements were supervised and analysis of this data considered up-to-date brand level information, including details on food fortification. The information was collected in early pregnancy to minimize the recall bias associated with the completion of questionnaires after childbirth.

A limitation of the study was that convenience recruitment was used rather than consecutive recruitment which is not feasible in a busy maternity service with a single interviewer. The information on smoking was self-reported and not validated by biochemical testing. However, any non-disclosures of smoking were analysed with the non-smokers and thus, it is likely that the dietary differences we found would be even greater if smoking could be verified by biochemical screening. Dietary information was gathered in the first trimester only and it is not known if dietary choices changed as pregnancy advanced. An Irish study of 285 women who completed a three-day food diary in each trimester used cluster analysis to identify two major dietary patterns, “Unhealthy” and “Health conscious”. Comparing the energy and nutrient intakes from the first trimester to the third trimester they found no significant change in either cluster. [33] It is also difficult to compare our findings on the relationship between dietary intakes and smoking behaviour in our study with previous

For peer review only

reports because surveys on dietary intakes in adults usually excluded women who were pregnant or breastfeeding. [21]

CONCLUSIONS

Our findings that pregnant women who continue to smoke have dietary inadequacies strengthens the public health case for screening women in early pregnancy and for offering support for smoking cessation. The dietary intakes of former smokers suggest that dietary intakes improve after smoking cessation. For those women that continue to smoke in pregnancy, it also highlights the need to advise women about healthy eating behaviours and about the need to start Folic Acid supplementation.

ACKNOWLEDGEMENTS

This work was supported by the all–island body Safefood. We also thank the Friends of the Coombe for their support. We thank the women who participated and our laboratory and midwifery colleagues for their assistance.

AUTHOR CONTRIBUTIONS

EOM contributed to the conception and design of the study, collected data, performed data analysis and wrote and edited the manuscript. SC conducted the primary study involving patient recruitment, issuing questionnaires, analysis of food diaries and gathering data and contributed to the conception and design of this study and drafting of the manuscript. RK, CR

For peer review only

and SC were involved in the conception and design of this study, data analysis and contributed to writing and editing. AM was involved in conception and design of the study, drafting sections of the manuscript and approving the final submitted version. MT contributed to the conception and design of the study, analysis of data and writing and editing of the manuscript.

FUNDING

This work was supported by the all island body *Safe*food (grant number 02-2015).

DECLARATION OF INTERESTS

None of the authors have any conflicts of interest to declare.

PATIENT CONSENT

Written consent was obtained.

ETHICAL APPROVAL

The study was approved by the Coombe Women and Infants University Hospital Research and Ethics Committee (REC) – reference number 27-2013.

DATA SHARING STATEMENT

Extra data is available by emailing eimer.om@gmail.com.

For peer review only

REFERENCES

1. Shea AK, Steiner M. Cigarette smoking during pregnancy. *Nicotine Tob. Res*. 2008;10:267-278
2. Cnattingius S. The epidemiology of smoking during pregnancy: smoking prevalence, maternal characteristics, and pregnancy outcomes. *Nicotine Tob Res*, 2004;6:125-140
3. Blatt K, Moore E, Chen A, et al. Association of Reported Trimester-Specific Smoking Cessation and Fetal Growth Restriction. *Obstet Gynecol* 2015;125:1452-1459.
4. Barker DJ, Winter PD, Osmond C, et al. Weight in infancy and death from ischaemic heart disease. *Lancet* 1989;2:577-580
5. Fall CH, Yajnik CS, Rao S, et al. Micronutrients and fetal growth. *J Nutr* 2003;133:1747-1756
6. Blumfield ML, Hure AJ, Macdonald-Wicks L, et al. Systematic review and meta-analysis of energy and macronutrient intakes in pregnancy in developed countries. *Nutr Rev* 2012;6:322-336
7. Fattah C, Farah N, O'Toole F, et al. Body Mass Index (BMI) in women booking for antenatal care: Comparison between selfreported and digital measurements. *Eur J Obstet Gynecol Reprod*. 2009;144:32–4.

8. Fattah, C, Farah, N, Barry, SC., et al. Maternal weight and body composition in the first trimester of pregnancy. *Acta Obstet Gynecol Scand* 2010;89(7):952-955.

9. McNulty B, Pentieva, K, Marshall, B, et al. Women's compliance with current folic acid recommendations and achievement of optimal vitamin status for preventing neural tube defects. *Hum Reprod*. 2011;26:1530-1536

10. Summary of dietary reference values – version 4 (September 2017) – European Food Safety Authority available at https://www.efsa.europa.eu/sites/default/files/2017_09_DRVs_summary_report.pdf Accessed on 21/2/2018

11. Goldberg GR, Black AE, Jebb SA, et al. Critical evaluation of energy intake data using fundamental principles of energy physiology: 1. Derivation of cut-off limits to identify under-recording. *Eur J Clin Nutr* 1991;45:569-581.

12. Black AE. Critical evaluation of energy intake using the Goldberg cut-off for energy intake: basal metabolic rate. A practical guide to its calculation, use and limitations. *Int J Obes Relat Metab Disord* 2000;24:1119-1130.

13. Central Statistics Office (CSO) (2013) Survey on income and living conditions, Cork, Ireland CSO. Available at: http://pdf.cso.ie/www/pdf/20170908023951_Survey_on_Income_and_Living_Conditions_2013_full.pdf
Accessed October 18, 2017

14. Coombe Women and Infants University Hospital Annual Clinical Report 2014. Available at: http://www.coombe.ie/index.php?nodeId=110. Accessed October 24, 2017

15. Dallongeville J, Marecaux N, Fruchart JC, et al. Cigarette smoking is associated with unhealthy patterns of nutrient intake: a meta-analysis. *J Nutr* 1998;128:1450-7.

For peer review only

16. Palaniappan U, Jacobs Starkey L, O'Loughlin J, et al. Fruit and vegetable consumption is lower and saturated fat intake is higher among Canadians reporting smoking. *J Nutr* 2001;131:1952-8.

17. Dyer AR, Elliot E, Stamler J, et al. (INTERMAP Research Group). Dietary intake in male and female smokers, ex-smokers, and never smokers: The INTERMAP Study. J *Hum Hypertens* 2003;17:641-54

18. Thompson RL, Margetts BM, Speller VM, et al. The Health Education Authority's health and fruit lifestyle survey 1993: who are the low fruit and vegetable consumers? *J Epidemiol Community Health* 1999;53:294-9.

19. Haste FM, Brooke OG, Anderson HR, et al. The effect of nutritional intake on outcome of pregnancy in smokers and non-smokers. *Br J Nutr* 1991;65:347-354

20. Mathews F, Yudkin P, Smith RF, et al. Nutrient intakes during pregnancy: the influence of smoking status and age. *J Epidemiol Community Health* 2000;54:17-23.

21. Subar AF, Harlan LC, Mattson ME. Food and nutrient intake differences between smokers and non-smokers in the US. *Am J Public Health* 1990;80:1323-9

22. Morabia A, Curtin F, Bernstein MS. Effects of smoking and smoking cessation on dietary habits of a Swiss urban population. *Eur J Clin Nutr*. 1999 Mar;53(3):239.

23. .Chelchowska M, Ambroszkiewicz J, Gajewska J, Laskowska-Kilta T and Leibschang J. The effect of tobacco smoking during pregnancy on plasma oxidant and antioxidant status in mother and newborn. *Eur J Obstet Gynecol Reprod Biol* 2011;155;132-136

24. Chéruel F, Jarleir M, Sancho-Garnier H. Effect of cigarette smoking on gustatory sensitivity, evaluation of the deficit and of recovery time course after smoking cessation. *Tob Induc Dis* 2017;15:15

For peer review only

25. King JC. Physiology of pregnancy and nutrient metabolism. *Am J Clin Nut* 2000;71:1218-1225.

26. Englund-Ogge L, Brantsaeter AL, Verena LS, et al. Maternal dietary patterns and preterm delivery: results from a large prospective cohort study. *BMJ* 2014;348:1446

27. Micronutrient needs during pregnancy and lactation. Drake VJ et al, updated 2016. Oregon state university. Report online. Available at: http://lpi.oregonstate.edu/mic/life-stages/pregnancy-lactation . Accessed on October 29th 2017

28. Mei-Dan E, Walfisch A, Weisz B, Hallak M, Brown R, Shrim A. The unborn smoker: association between smoking during pregnancy and adverse perinatal outcomes. Journal of perinatal medicine. 2015 Sep 1;43(5):553-8.

29. Wang M, Wang ZP, Gong R, et al. Maternal smoking during pregnancy and neural tube defects in offspring: a meta-analysis. *Childs Nerv Syst* 2013;30:83-89

30. Daly LE, Kireke PN, Molloy A, et al. Folate levels and neural tube defects-implications for prevention *JAMA* 1995;274:1698-1702

31. Edmond JA, Karagas MR, Baker ER, et al. Better Diet Quality during Pregnancy Is Associated with a Reduced Likelihood of an Infant Born Small for Gestational Age: An Analysis of the Prospective New Hampshire Birth Cohort Study. *J Nutr.* 2018;148(1):22-30.

32. Chatzi L, Mendez M, Garcia R et al. Mediterranean diet adherence during pregnancy and fetal growth: INMA (Spain) and RHEA (Greece) mother-child cohort studies. *Br J Nutr*. 2012;107(1):135-45.

33. McGowan CA, McAuliffe FM. Maternal dietary patterns and associated nutrient intakes during each trimester of pregnancy. *Public Health Nutr*. 2013;16(1):97-107

For peer review only

Table 1. Characteristics of the study sample including delivery outcomes

	Study sample (n=502)[a]	Women with dietary data (n=398)	Women without dietary data (n=104)	P
Age (years) (mean ± SD)	30.7 ± 5.5	30.5 ± 5.6	31.2 ± 5.0	NS
BMI (kg/m^2) (mean ± SD)	26.0 ± 5.5	25.9 ± 5.4	26.1 ± 5.8	NS
Obese (%)	19.3% (97)	19.2% (76)	20.1%(21)	NS
Nulliparous (%)	42.6% (214)	45.5% (181)	31.7% (33)	0.012
Irish-born (%)	70.1% (352)	76.9% (306)	44.2% (46)	<0.001
Alcohol intake during pregnancy (%)	4.0% (20)	4.8 % (19)	0.9% (1)	NS
Taking FA pre-pregnancy	42.8% (215)	43.2% (172)	41.3% (43)	NS
Birth weight (grams) (mean +/- SD)	3422 ± 573.3	3426.9 ±586.1	3402.5 ±520.9	NS

(significance level – 0.05, statistical tests used; chi-square test for categorical variables and independent samples t-test for continuous variables)

For peer review only

[a] Of the 502 women studied, delivery outcomes were available on 472; 378 with dietary data and 94 without dietary data. The remaining women delivered elsewhere.

NS – not significant, FA – Folic acid, SD – Standard Deviation, BMI – Body Mass Index, kg – Kilogram

Table 2. Comparison of characteristics between smokers and non-smokers with dietary data available (n=398)

	All women (n=398)[a]	Smokers (n=50)	Non-smokers (n=348)[b]	p
Age (years) (mean ± SD)	30.5 ± 5.6	27.0 ± 6.44	31.0 ± 5.3	<0.001
BMI (kg/m^2) (mean ± SD)	25.9 ±5.4	25.3 ± 4.7	26.0 ±5.5	0.418
Obese (%)	19.2% (76)	22% (11)	18.7% (65)	0.323
Nulliparous (%)	45.5% (181)	46.0% (23)	45.4% (158)	0.937
Irish-born (%)	76.9% (306)	82% (41)	76.1% (265)	0.739
Alcohol intake during pregnancy (%)	4.8 % (19)	6.1% (3)	4.7% (16)	0.863
Birth weight (grams)	3426.9 ±	3134.4 ± 622.1	3469.46 ±	<0.001

For peer review only

(mean ± SD)	586.1	569.2

(significance level – 0.05, statistical tests used – chi-square test for categorical variables and independent samples t-test for continuous variables)

[a] Of the 398 women studied, delivery outcomes were available on 378; 330 who were non-smokers and 48 who were current smokers. The remaining women delivered elsewhere.

[b] As dietary composition was similar between never smokers and former smokers (Supplementary tables 1 and 2) they are combined into one "non-smokers" group.

FA – folic acid, SD – standard deviation, BMI – Body Mass Index

For peer review only

Table 3. Daily macronutrient and micronutrient intakes in smokers compared to non-smokers

	Smokers (n=50) (median, IQ range)	Non-smokers (n=348)[a] (median, IQ range)	p
Protein (g)	69.1 (31.3)	74.4 (28.0)	0.168
Carbohydrate (g)	204.3 (95.7)	198.8 (73.7)	0.820
Fat (g)	75.5 (42.8)	72.7 (39.1)	0.421
Saturates (g)	30.3 (15.1)	27.2 (15.7)	0.353
Monounsaturated fat (g)	25.3 (13.7)	24.5 (13.6)	0.928
Polyunsaturated fat (g)	8.8 (7.0)	10.3 (7.0)	0.176
Fibre (g)	13.1 (7.7)	16.3 (8.5)	<0.001
Sodium (mg)	2100.7 (1158.7)	1877.0 (971.9)	0.095
Potassium (mg)	2371.2 (1085.7)	2533.1 (1038.5)	0.305
Calcium (mg)	797.8 (538.06)	788.4 (536.4)	0.601
Magnesium (mg)	196.0 (75.8)	226.9 (100.5)	0.003
Phosphorous (mg)	1060.5 (381.7)	1180.8 (490.1)	0.063
Iron (mg)	7.9 (3.9)	9.5 (4.25)	0.002

For peer review only

Copper (mg)	0.7 (0.4)	0.9 (0.5)	0.001
Zinc (mg)	7.1 (3.9)	7.8 (3.7)	0.227
Chloride (mg)	3187.3 (1678.0)	2843.7 (1345.8)	0.067
Iodine (µg)	91.5 (89.5)	104.5 (77.0)	0.500
Retinol (µg)	312.6 (225.0)	282.9 (242.3)	0.916
Carotene (µg)	1488.3 (3427.4)	3213.2 (4479.5)	0.004
Vitamin D (µg)	2.3 (2.5)	2.4 (2.6)	0.547
Vitamin C (mg)	71.6 (74.3)	83.8 (83.0)	0.035
Vitamin E (µg)	7.1 (5.1)	8.2 (5.6)	0.196
Thiamine (mg)	1.3 (0.6)	1.4 (0.7)	0.100
Riboflavin (mg)	1.3 (0.9)	1.4 (0.7)	0.040
Niacin (mg)	30.5 (16.8)	34.1 (14.4)	0.089
Vitamin B6 (mg)	1.7 (1.0)	1.9 (0.9)	0.451
Vitamin B12 (µg)	4.0 (3.5)	4.0 (2.6)	0.767
Folate (natural and fortified) (µg)	202.1 (127.3)	241.9 (142.8)	0.006

(significance level – 0.05, statistical tests used; Kruskal-Wallis)

[a] As dietary composition was similar between never smokers and former smokers (Supplementary tables 1 and 2) they are combined into one "non-smokers" group.

SD – standard deviation, IQ range – Interquartile range, g – grams, mg – milligrams, µg - micrograms

For peer review only

Table 4. Total median (IQR) maternal dietary folate (including fortified folic acid) per day in early pregnancy and hematological measures of folate in non-smokers compared to current smokers (n=398).

	Non-smokers (n=348)[a]	Smokers (n=50)	p
Natural dietary folate intake (mcg)	193.9 (93.9)	174.1 (112.7)	0.029
Fortified dietary folate intake (mcg)	33.8 (50.7)	16.9 (59.4)	0.076
Total dietary folate intake (mcg)	241.9 (142.8)	202.1 (127.1)	0.006
Dietary folate equivalent (mcg)	268.2 (190.7)	214.6 (146.9)	0.005
Achieving WHO DFE for pregnancy (>/= 600 mcg DFE) (%, n)	6.3 % (22)	6.0 % (3)	0.930
Achieving WHO DFE for lactation (>/= 500 mcg DFE) (%,n)	10.9 % (38)	6.0 % (3)	0.285
Pre-pregnancy FA use (%,n)	48.0 % (167)	10.0 % (5)	0.015

For peer review only

Serum folate (nmol/L), (median, IQR)	35.2 +/- 17.0	22.3 +/- 19.5	<0.001
Red blood cell (RBC) folate (nmol/L), (mean, SD)	1195.1 +/- 440.0	820.0 +/- 362.5	<0.001
Achieving optimal RBC folate (%,n)[b]	71.3 % (248)	38.0 % (19)	<0.001

(significance level – 0.05, statistical tests used – chi-square test for categorical variables and independent samples t-test for continuous variables (normally distributed) and Kruskal-Wallis for comparison of non-normally distributed variables)

IQR– interquartile range, mcg – micrograms, WHO – World Health Organization, SD – standard deviation

DFE – Dietary folate equivalent. For natural folate from food – 1mcg folate from food is equal to 1mcg DFE. For food fortified with folate 1mcg of folate = 1.7 DFE, as natural folate from food is 50% bioavailable compared to 85% bioavailability from fortified folate and folate supplementation (85/50 = 1.7)

[a] As dietary composition was similar between never smokers and former smokers (Supplementary tables 1 and 2) they are combined into one "non-smokers" group.

[b] The reported optimal threshold concentration for red blood cell folate is reported as 906nmol/L by Daly et al.[30] At and above this concentration, the risk of neural tube defects (NTDs) was reduced to <8 NTDs/10,000 live births.

For peer review only

Supplementary Table 1:

Comparison of macronutrient intake between never smokers and former smokers

	Never smokers (n=193) (median (IQ range))	Former smokers (n=155) (median (IQ range))	P
Carbohydrate (g)	199.0 (68.2)	197.0 (89.6)	0.535
Protein (g)	72.8 (26.0)	77.0 (29.1)	0.027
Fat (g)	68.8 (40.1)	77.8 (36.4)	0.071
Saturated fat (g)	25.6 (14.2)	28.5 (16.3)	0.211
Monounsaturated fat (g)	24.0 (14.2)	25.5 (12.8)	0.256
Polyunsaturated fat (g)	10.1(7.2)	10.7 (7.3)	0.179
Fibre (g)	16.3 (8.8)	16.2 (8.0)	0.574

(Significance level, 0.05. Statistical test used – Kruskal-Wallis)

IQ range – interquartile range, g – grams

For peer review only

Supplementary Table 2: Comparison of micronutrient intake between never smokers and former smokers

	Never smokers (n=193) (median (IQ range))	Former smokers (n=155) (median (IQ range))	P
Sodium (mg)	1805.2 (928.7)	2069.6 (988.1)	0.008
Potassium (mg)	2472.9 (946.4)	2631.2 (1127.0)	0.102
Chloride (mg)	2688.6 (1294.4)	3067.6 (1329.7)	0.045
Calcium (mg)	749.0 (505.8)	838.0 (533.0)	0.120
Phosphorous (mg)	1124.5 (461.6)	1212.2 (463.5)	0.055
Iron (mg)	9.1 (3.5)	10.0 (5.5)	0.014
Magnesium (mg)	225.1 (98.0)	229.8 (110.3)	0.334
Zinc (mg)	7.7 (3.8)	8.1 (3.9)	0.041
Copper (mg)	0.9 (0.5)	0.9 (0.5)	0.501
Iodine (mcg)	101.8 (69.8)	106.9 (86.7)	0.080
Retinol (mcg)	274.6 (248.2)	310.9 (231.8)	0.089
Carotene (mcg)	3475.6 (5193.8)	2965.4 (4199.2)	0.300
Vitamin C (mg)	79.9 (88.5)	89.5 (78.2)	0.174
Vitamin D (mcg)	2.2 (2.5)	2.5 (2.9)	0.438
Vitamin E (mcg)	8.0 (5.5)	8.4 (5.5)	0.123

For peer review only

Thiamine B1 (mg)	1.4 (0.7)	1.4 (0.6)	0.293
Riboflavin B2 (mg)	1.4 (0.7)	1.6 (0.8)	0.008
Niacin B3 (mg)	33.3 (14.1)	35.8 (15.0)	0.038
Vitamin B6 (mg)	1.9 (0.8)	1.9 (0.8)	0.191
Vitamin B12 (mg)	3.8 (2.8)	4.1 (2.6)	0.018
Folic Acid (mcg)	234.1 (135.9)	249.0 (148.9)	0.154

(Significance level, 0.05. Statistical test used – Kruskal-Wallis)

IQ – interquartile range, mg – milligrams, mcg - micrograms

For peer review only

Supplementary Table 3: Comparison of macronutrient intakes between smokers and non-smokers (excluding likely energy under-reporters i.e. plausible reporters only)

	Non-smokers (n=222) (median (IQ range))[a]	Current smokers (n=34) (median (IQ range))	P
Carbohydrate (g)	217.3 (72.8)	230.0 (94.2)	0.229
Protein (g)	80.2 (25.1)	78.3 (33.5)	0.566
Fat (g)	84.3 (33.0)	90.6 (34.8)	0.416
Fibre (g)	18.3 (8.1)	14.6 (6.3)	0.004
Saturated fat (g)	31.5 (16.6)	35.5 (13.4)	0.472
Monounsaturated fat (g)	29.0 (12.5)	27.9 (12.8)	0.978
Polyunsaturated fat (g)	12.1 (7.3)	10.8 (8.3)	0.236

(Significance level, 0.05. Statistical test used – Kruskal-Wallis)

[a] As dietary composition was similar between never smokers and former smokers (Supplementary tables 1 and 2) they are combined into one "non-smokers" group.

IQ range – interquartile range, g - grams

For peer review only

Supplementary Table 4: Comparison of micronutrient intakes between smokers and non-smokers (excluding likely energy under-reporters i.e. plausible reporters only)

	Non-smokers (n=222) (median (IQ range))[a]	Current smokers (n=34) (median (IQ range))	P
Sodium (mg)	2126.2 (991.6)	2499.4 (1092.7)	0.079
Potassium (mg)	2785.8 (965.7)	2764.8 (1037.3)	0.631
Chloride (mg)	3156.8 (1399.0)	3624.4 (1747.9)	0.031
Calcium (mg)	961.9 (589.9)	848.8 (393.2)	0.396
Phosphorous (mg)	1299.2 (451.4)	1137.7 (479.4)	0.113
Iron (mg)	10.4 (4.3)	9.7 (3.7)	0.023
Magnesium (mg)	252.4 (101.0)	214.7 (80.8)	0.012
Zinc (mg)	8.6 (3.4)	8.1 (3.8)	0.357
Copper (mg)	1.0 (0.5)	0.8 (0.3)	0.009
Iodine (mcg)	120.5 (84.9)	124.8 (92.1)	0.889
Retinol (mcg)	344.9 (282.7)	344.2 (248.9)	0.836
Carotene (mcg)	3213.2 (4762.3)	1488.3 (3572.3)	0.016
Vitamin C (mg)	97.0 (90.8)	77.7 (69.6)	0.097
Vitamin D (mcg)	2.6 (2.9)	2.7 (2.5)	0.726
Vitamin E (mcg)	9.2 (5.4)	8.6 (7.2)	0.321

For peer review only

Thiamine B1 (mg)	1.5 (0.6)	1.5 (0.6)	0.486
Riboflavin B2 (mg)	1.7 (0.8)	1.5 (1.0)	0.244
Niacin B3 (mg)	36.1 (13.7)	33.4 (15.7)	0.562
Vitamin B6 (mg)	2.0 (0.8)	2.1 (0.8)	0.645
Vitamin B12 (mg)	4.5 (2.7)	5.1 (3.9)	0.528
Folic Acid (mcg)	266.2 (123.8)	213.4 (135.9)	0.009

(Significance level, 0.05. Statistical test used – Kruskal-Wallis)

[a] As dietary composition was similar between never smokers and former smokers (Supplementary tables 1 and 2) they are combined into one "non-smokers" group.

IQ range – interquartile range, mg – milligramsmcg – micrograms

For peer review only

STROBE 2007 (v4) checklist of items to be included in reports of observational studies in epidemiology*
Checklist for cohort, case-control, and cross-sectional studies (combined)

Section/Topic	Item #	Recommendation	Reported on page #
**Title and abstract**	1	(*a*) Indicate the study's design with a commonly used term in the title or the abstract	1
		(*b*) Provide in the abstract an informative and balanced summary of what was done and what was found	2
**Introduction**			
Background/rationale	2	Explain the scientific background and rationale for the investigation being reported	5
Objectives	3	State specific objectives, including any pre-specified hypotheses	5
**Methods**			
Study design	4	Present key elements of study design early in the paper	5-6
Setting	5	Describe the setting, locations, and relevant dates, including periods of recruitment, exposure, follow-up, and data collection	2,5-6
Participants	6	(*a*) *Cohort study*—Give the eligibility criteria, and the sources and methods of selection of participants. Describe methods of follow-up<br>*Case-control study*—Give the eligibility criteria, and the sources and methods of case ascertainment and control selection. Give the rationale for the choice of cases and controls<br>*Cross-sectional study*—Give the eligibility criteria, and the sources and methods of selection of participants	5-6
		(*b*) *Cohort study*—For matched studies, give matching criteria and number of exposed and unexposed<br>*Case-control study*—For matched studies, give matching criteria and the number of controls per case	n/a
Variables	7	Clearly define all outcomes, exposures, predictors, potential confounders, and effect modifiers. Give diagnostic criteria, if applicable	2
Data sources/ measurement	8*	For each variable of interest, give sources of data and details of methods of assessment (measurement). Describe comparability of assessment methods if there is more than one group	5-7
Bias	9	Describe any efforts to address potential sources of bias	6-7
Study size	10	Explain how the study size was arrived at	2
Quantitative variables	11	Explain how quantitative variables were handled in the analyses. If applicable, describe which groupings were chosen and why	6-7
Statistical methods	12	(*a*) Describe all statistical methods, including those used to control for confounding	7
		(*b*) Describe any methods used to examine subgroups and interactions	7
		(*c*) Explain how missing data were addressed	8
		(*d*) *Cohort study*—If applicable, explain how loss to follow-up was addressed<br>*Case-control study*—If applicable, explain how matching of cases and controls was addressed	n/a

For peer review only

		Cross-sectional study—If applicable, describe analytical methods taking account of sampling strategy	
		(*e*) Describe any sensitivity analyses	n/a
**Results**			
Participants	13*	(a) Report numbers of individuals at each stage of study—eg numbers potentially eligible, examined for eligibility, confirmed eligible, included in the study, completing follow-up, and analysed	8
		(b) Give reasons for non-participation at each stage	8
		(c) Consider use of a flow diagram	n/a
Descriptive data	14*	(a) Give characteristics of study participants (eg demographic, clinical, social) and information on exposures and potential confounders	8, 21-22
		(b) Indicate number of participants with missing data for each variable of interest	Footnotes of tables
		(c) *Cohort study*—Summarise follow-up time (eg, average and total amount)	n/a
Outcome data	15*	*Cohort study*—Report numbers of outcome events or summary measures over time	n/a
		Case-control study—Report numbers in each exposure category, or summary measures of exposure	n/a
		Cross-sectional study—Report numbers of outcome events or summary measures	24,25,26
Main results	16	(*a*) Give unadjusted estimates and, if applicable, confounder-adjusted estimates and their precision (eg, 95% confidence interval). Make clear which confounders were adjusted for and why they were included	n/a
		(*b*) Report category boundaries when continuous variables were categorized	27
		(*c*) If relevant, consider translating estimates of relative risk into absolute risk for a meaningful time period	n/a
Other analyses	17	Report other analyses done—eg analyses of subgroups and interactions, and sensitivity analyses	Supplementary tables
**Discussion**			
Key results	18	Summarise key results with reference to study objectives	8,9
Limitations	19	Discuss limitations of the study, taking into account sources of potential bias or imprecision. Discuss both direction and magnitude of any potential bias	14,14
Interpretation	20	Give a cautious overall interpretation of results considering objectives, limitations, multiplicity of analyses, results from similar studies, and other relevant evidence	10-14
Generalisability	21	Discuss the generalisability (external validity) of the study results	14
**Other information**			
Funding	22	Give the source of funding and the role of the funders for the present study and, if applicable, for the original study on which the present article is based	15,16

*Give information separately for cases and controls in case-control studies and, if applicable, for exposed and unexposed groups in cohort and cross-sectional studies.

Note: An Explanation and Elaboration article discusses each checklist item and gives methodological background and published examples of transparent reporting. The STROBE checklist is best used in conjunction with this article (freely available on the Web sites of PLoS Medicine at http://www.plosmedicine.org/, Annals of Internal Medicine at http://www.annals.org/, and Epidemiology at http://www.epidem.com/). Information on the STROBE Initiative is available at www.strobe-statement.org.

For peer review only

BMJ Open

Comparison at the first prenatal visit of the maternal dietary intakes of smokers with non-smokers in a large maternity hospital: a cross sectional study.

Journal:	*BMJ Open*
Manuscript ID	bmjopen-2018-021721.R2
Article Type:	Research
Date Submitted by the Author:	17-May-2018
Complete List of Authors:	O'Malley, Eimer<br>Cawley, Shona; University College Dublin, UCD Centre for Human Reproduction; Dublin Institute of Technology<br>Reynolds, Ciara; University College Dublin, UCD Centre for Human Reproduction<br>Kennedy, Rachel; University College Dublin, UCD Centre for Human Reproduction; Dublin Institute of Technology<br>Molloy, Anne; University of Dublin Trinity College, School of Medicine<br>Turner, Michael; University College Dublin
**Primary Subject Heading**:	Obstetrics and gynaecology
Secondary Subject Heading:	Nutrition and metabolism
Keywords:	Fetal medicine < OBSTETRICS, Maternal medicine < OBSTETRICS, PUBLIC HEALTH

SCHOLARONE™ Manuscripts

Title: Comparison at the first prenatal visit of the maternal dietary intakes of smokers with non-smokers in a large maternity hospital: a cross sectional study

Authors: O'Malley EG[1], Cawley S[1,2], Reynolds CME[1], Kennedy RAK[1,2], Molloy A[3], Turner MJ[1].

Affiliations [1]UCD Centre for Human Reproduction, Coombe Women and Infants University Hospital, Cork St, Dublin 8, Ireland

[2] School of Biological Sciences, Dublin Institute of Technology, Kevin St., Dublin 8, Ireland

[3] School of Medicine, Trinity College Dublin, Dublin 2, Ireland.

Corresponding Author: E O'Malley

Email: eimer.om@gmail.com

Telephone: +35314085571

Key words: Maternal dietary intakes, Maternal smoking, Intrauterine fetal growth, Small for gestational age

Word Count: 2840 words

For peer review only

ABSTRACT

- **Objectives:** Using detailed dietary and supplement questionnaires in early pregnancy we aimed to compare the dietary intakes of micronutrients and macronutrients at the first prenatal visit of women who reported continuing to smoke during pregnancy with the intakes of women who were non-smokers.
- **Design:** Cross sectional study conducted between June 2014 and March 2016.
- **Setting:** Stand-alone tertiary maternity hospital in an urban setting with approximately 8000 deliveries/ year
- **Participants:** Women were recruited at their convenience after sonographic confirmation of an ongoing singleton pregnancy (n=502). Detailed dietary and supplement information was available for 398 women. Women <18 years and those who did speak English fluently were excluded.
- **Primary and secondary outcome measures:** The differences in dietary micronutrients and macronutrients and maternal folate levels between women who continued to smoke in pregnancy compared to non-smokers.
- **Results:** Of the 502 women, the mean age was 30.5 (SD 5.6) years, 42.5% were nulliparas, 19.2% were obese and 398 (79.3%) completed the questionnaire satisfactorily. In the 50 (12.6%) current smokers, the micronutrients magnesium, iron, carotene and copper were lower (all p<0.005) whereas sodium and chloride were higher compared with the 348 (87.4%) non-smokers. Smokers reported lower intakes of dietary total folate (p=0.006) compared with non-smokers (i.e. dietary folate equivalents; intake from natural and fortified dietary sources) (p=0.005). Smokers also reported lower intakes of fibre than non-smokers (13.1g (IQR 7.7) vs 16.3g (IQR 8.5), p<0.001). The dietary intakes of former smokers compared favourably with non-smokers.

For peer review only

- **Conclusions:** We found that women who continue to smoke during pregnancy have serious dietary inadequacies which potentially may aggravate fetal growth restriction associated with direct toxicity from cigarettes. This provides a further reason to promote smoking cessation interventions in pregnancy, but also highlights the need for dietary and supplementation interventions in women who continue to smoke.
- **Trial registration:** n/a

Word count = 298/300

For peer review only

Strengths and limitations of this study

- This study was a cross-sectional study with a well characterised study sample.
- The detailed dietary and supplement questionnaires were supervised and analysis of this data included up to date brand level information including details on food fortification with folate.
- Collection of the information in early pregnancy was conducted to minimise the recall bias associated with completion of the questionnaires after delivery.
- A limitation of the study is that convenience recruitment was used rather than consecutive recruitment, as the later was not possible with a single interviewer.
- The information on smoking in this study was self-reported and not validated by biochemical testing. However, any non-disclosers would be analysed with the non-smokers and it is likely that the dietary differences we found would be even greater if smoking could be verified by biochemical screening.

For peer review only

INTRODUCTION

Maternal smoking is strongly associated with an increase in adverse pregnancy outcomes including miscarriage, preterm delivery, congenital malformations and stillbirth. [1,2] In particular, it is associated with an increased risk of intrauterine fetal growth restriction (IUGR) and the risk increases with the number of cigarettes smoked. Smoking is an important modifiable risk factor for IUGR because cessation of smoking in the first half of pregnancy may normalize fetal growth. [3] Yet, many women continue to smoke throughout pregnancy.

Fetal growth and lifelong pregnancy outcomes are also influenced by maternal nutrition. [4,5] Deficiencies of both maternal micronutrients and macronutrients have been associated with aberrant fetal growth. In a systematic review and meta-analysis of 62 studies from developed countries, pregnant women had dietary folate, iron and vitamin D dietary intakes consistently below nutrient recommendations. [6] Forty-one of the studies, however, did not report maternal smoking status which may influence dietary intakes.

This cross-sectional study compared the dietary intakes of micro- and macronutrients of self-reported current smokers with the intakes of non-smokers at the first antenatal visit in a large maternity hospital.

METHODS

Women were recruited at their convenience after sonographic confirmation of an ongoing singleton pregnancy during their first prenatal visit between June 2014 and March 2016. Women with multiple pregnancies or women who did not speak English fluently were excluded. Clinical and sociodemographic details were computerized routinely by a trained midwife using a standard questionnaire and a barcode system. The

For peer review only

hospital questionnaire included questions on whether the woman had never smoked, had stopped smoking before her first visit or was continuing to smoke. The number of cigarettes smoked per day was also recorded as 1-5/day, 6-10/day or >10/day. The maternal weight and height were measured by a midwife at the first antenatal visit and the Body Mass Index (BMI) was calculated and categorised. This is more accurate than a self-reported pre-pregnancy weight. [7] Body weight, BMI and body composition have been shown to not vary significantly across the first trimester in a study of 1000 women.[8] Written informed consent was obtained.

All women were asked to complete a retrospective four-day diet history (DH) at the first antenatal visit. This was combined with a previously validated food frequency questionnaire (FFQ) which had been customized to capture food fortification with folate at brand level in Ireland.[9]Quantitative food intake data from the DH was divided by four to derive the average daily intake for each food. These data were entered into Nutritics Version 3.7 software to convert the food intakes into nutrient measurements. The nutrient requirement reference levels for pregnancy (either average requirement, population reference intake or adequate intake) were obtained from the European Food Safety Authority (EFSA) guideline [10]. Levels for Sodium, Chloride and Vitamin E are not established.

To overcome the possible limitation associated with nutritional surveys of under-reporting, a calculation was applied to identify any likely under-reporters which is based on energy intake, basal metabolic rate and physical activity level.[11,12] Based on the reported physical activity level (PAL), the lowest plausible PAL threshold (from 1.45-2.20) was determined. If the energy intake (EI) to basal metabolic rate (BMR) ratio fell below the plausible threshold for their PAL then they were identified as a dietary under-reporter. Plausible energy intake reporters could be identified in this way.

For peer review only - http://bmjopen.bmj.com/site/about/guidelines.xhtml

Preliminary analyses conducted demonstrated that dietary composition was similar between never smokers and former smokers (Supplementary tables 1 and 2), therefore, analyses combined never and former smokers into one “non-smokers” group.

Information on socioeconomic status was analysed using questions from the European Union Survey on Income and Living Conditions (EU SILC). [13] All women had a full blood count taken and other biomarkers including serum folate, whole blood folate and red blood cell (RBC) folate levels were obtained at the same visit. Information on birth outcomes were obtained from the hospital computerized database which is updated immediately after delivery.

Data from the questionnaires were anonymised and entered into Microsoft Excel and exported for statistical analysis using SPSS Version 20 (IBM Corporation, Armonk, New York). Continuous data were assessed for normality by visual inspection of the histogram and box-plot and calculation of the kurtosis and skewness. Descriptive statistics were used to describe the characteristics of the study sample. The Kruskal-Wallis test for non-parametric data was used to assess the difference between nutrient intakes in women who were current smokers compared to non-smokers. Tests for statistical significance were performed with the level for significance set at $p<0.05$. The study was approved by the Hospital's Research Ethics Committee.

Patient and Public Involvement

The authors were aware of the increased risk of adverse pregnancy outcomes associated with maternal smoking and also the association of smoking with poor dietary quality compared to non-smokers. This was the stimulus for our research question.

The patients were not involved in the study design or the recruitment process however the Hospital Research Ethics Committee includes members of the public involved in reviewing the methodology, patient information leaflets and questionnaires.

For peer review only

The results of our study were not directly disseminated to the study population. They will be presented locally to educate staff on our findings with the aim of changing practice to benefit our patients.

RESULTS

Of the 502 women recruited, 398 (79.3%) completed the dietary questionnaire satisfactorily. The study sample was similar to the Hospital population. [14] Women cited time constraints as the main reason for not completing the dietary questionnaire and those who did fill out the dietary questionnaire were more likely to be nulliparous (p=0.012) and Irish-born (p<0.001) . Comparing current smokers to non-smokers, they were younger (p<0.001) with a higher percentage of relative income poverty compared to the non-smokers (non-smokers; 21.6% (40/185) vs. current smokers; 56.5% (13/23), p=0.009). As expected, the mean birth weight was lower in smokers compared with non-smokers (Table 1).

Table 2 presents the dietary intakes overall and stratified by smoking status. The micronutrients magnesium, iron, carotene, copper, vitamin C and riboflavin were lower in smokers compared with non-smokers but the micronutrients sodium and chloride were higher. Compared with non-smokers, women who smoked had lower levels of dietary folate (p = 0.006, Table 2) and were less likely to take pre-pregnancy folic acid (p<0.001, Table 3). Women who smoked had a lower fibre intake compared with non-smokers (p<0.001) (Table 2).

There was no relationship between the reported number of cigarettes smoked per day (1-5 cigarettes/day compared to ≥ 6 cigarettes/day) and macronutrient and micronutrient intakes.

Comparing the dietary intakes of former smokers (n=155) with never smokers (n=193), the former smokers had superior intakes of the micronutrients iron, zinc,

For peer review only

vitamin B12, vitamin B2 (riboflavin) and Vitamin B3 (niacin) (Supplementary Tables 1 and 2). They had higher intakes in protein, sodium and chloride.

Table 3 compares the dietary folate intakes for smokers with non-smokers. Non-smokers reported a greater total dietary folate intake (p=0.006) with a superior intake of natural dietary folate compared to smokers (median 193.9 mcg (interquartile range (IQR) 93.9) vs median 174.1 mcg (IQR 174.1) (p=0.029)). However, both groups had a low percentage of women meeting the World Health Organization (WHO) dietary folate equivalent recommended for pregnancy (non-smokers; 6.3% vs smokers; 6.0%) and breastfeeding (non-smokers; 10.9% vs smokers; 6%). This dietary information is supported by the maternal folate measured at the first prenatal visit. Non-smokers had higher serum folate levels (35.2 ± 17.0 nmol/L vs 22.3 ± 19.5nmol/L) and red cell folate levels (1195.1 ± 440.0 nmol/L vs. 820.0 ± 362.5 nmol/L) with 71.3% (248) of the non-smokers achieving the optimal RBC folate level of 906 nmol/L compared with only 38.0% (19) of the smokers.

Of the 348 women in the non-smoking group, 63.8% (222) were plausible energy intake reporters compared to 68% (34) of the women in the current smoker group. Comparing the dietary intakes including any plausible energy intake reporters, lower intakes of fibre (p= 0.004), iron, copper, magnesium, carotene (all p<0.05) and folic acid (p=0.009) and a higher chloride intake (p<0.05) remained in the current smokers compared with non-smokers (Supplementary Tables 3 and 4).

For peer review only

DISCUSSION

Based on self-reporting at the first prenatal visit, we found that women who continued to smoke during pregnancy were more likely to have deficiencies of both dietary micronutrients and macronutrients than women who did not smoke. Interestingly, women who had stopped smoking had dietary intakes which compared favourably with women who never smoked.

Maternal smoking is strongly associated with adverse clinical outcomes for both the woman and her offspring and this study highlights that dietary quality and deficiencies are another area of concern. Our findings that smokers have a poor dietary composition relative to non-smokers is consistent with many previous studies, including those among the general adult population [15-18] and pregnant women [19,20].

Data from 11,260 adults aged 19-74 years from the second National Health and Nutrition Examination Survey (NHANES) were analyzed to determine food and nutrient differences between smokers and non-smokers. [21] In smokers, there was a lower intake of Vitamins A and C, folate and fibre and the intakes decreased as cigarette consumption increased. Smokers were less likely to consume fruit and vegetables, high fibre grains, low fat milk as well as vitamin and mineral supplements. These findings raised concerns that the lifelong risk of cancer was increased not only by cigarette smoking but also by dietary deficiencies.

A Swiss population study of 2301 men and 2306 women reported that current heavy smokers consumed less total vegetable proteins, carbohydrates, fibre, betacarotene, fruit and vegetables compared to non-smokers (p values all significant). [22] They also drank more alcohol and coffee ($p<0.0001$ and $p<0.005$ respectively). The female smokers also consumed less complex carbohydrates ($p<0.002$) and less iron ($p<0.02$). As in this study, the diet of ex-

For peer review only

smokers was similar to never smokers. This demonstrates that it is likely that quitting smoking is associated with other positive lifestyle modifications including improvement of diet.

A study comparing 60 smokers to 80 non-smokers in pregnancy demonstrated the oxidative stress associated with smoking through elevated levels of the oxidative malondialdehyde and corresponding lower levels of known antioxidants such as vitamin E, vitamin A and beta carotene in the plasma of smokers and cord blood of their infants compared to non-smokers and their infants.[23] Smoking produces free radicals that deplete antioxidant levels and these effects could be amplified by poor dietary intake of these important micronutrients.

The reasons for the differences in the dietary intakes between smokers and non-smokers are generally unexplained. One factor that may contribute is the impact of smoking on taste perception. A study conducted with 83 smokers and 48 non-smokers (male and female) using electrogustometry detected taste disturbance in the smokers that was dependent on the intensity of smoking. A follow-up of 24 of the smokers who quit smoking demonstrated a rapid recovery in taste sensitivity within two weeks in certain loci of the tongue and up to 12 months at other sites.[24] The daily requirement for many micronutrients increases in pregnancy to meet the higher physiologic demands. [25] There are both short and long term consequences of fetal undernutrition. A Norwegian prospective cohort study of 66,000 pregnant women conducted in 2002-8 used a food frequency questionnaire (FFQ) to assess dietary habits and established three patterns: “prudent” (vegetables, fruits, oils, water as beverage, whole grain cereals and fibre rich bread), “western” (salty and sweet snacks, white bread, desserts and processed meats) and “traditional” (potatoes, fish). After adjustment for covariates, higher scores for the “prudent” dietary choices were associated with a decreased risk of preterm delivery (0.88, 95% CI 0.80-

For peer review only - http://bmjopen.bmj.com/site/about/guidelines.xhtml

0.97). However, there was no independent association with the “western” diet pattern and preterm delivery. It was concluded that low adherence to the “prudent” pattern is a stronger indicator of unhealthy diet than intake of the western diet. [26]

Maternal smoking is associated with higher rates of small for gestational age infants but fetal growth retardation can extend beyond that cut-off point e.g. when an infant born at 3kg fails to reach their growth potential of 3.5kg. Fetal growth is dependent upon their nutrient uptake and the start point of this process is the maternal intake. A study based in India from 1994-7 of 797 pregnant women studied maternal diet and neonatal outcomes. There was a strong correlation between consumption of green leafy vegetables (GLVs), milk and fruit with a higher birth weight. For GLVs, it was shown that women who consumed them on alternate days delivered infants almost 200g heavier than those who never ate them. Furthermore, there were correlations between serum vitamin C and RBC folate levels and birth weight. [5] Considering the long-term consequences of undernutrition, the implications of this on the fetus are described by Barkers hypothesis with an increase in Type 2 diabetes mellitus, hypertension and coronary heart disease.[4,27]

A Canadian study conducted from 2001-7 of 1,646 self-reported smokers compared to 19,292 non-smokers, reported an increase in adverse perinatal and neonatal outcomes amongst smokers. There was an increased risk of preterm delivery (22.2% vs 12.4%, $p<0.05$), intrauterine fetal demise (OR 2.4 (95% CI 1.4-4.2), a mean birth weight approximately 200 grams lower in the current smokers ($p<0.05$) and an increased risk of neonatal death (1.2% vs 0.6, $p<0.05$). [28]

A systematic review and meta-analysis of 13 articles found a pooled odds ratio (OR) of NTDs in offspring of smokers of 1.03 (95% CI =0.80-1.33). When subgroup analysis was performed based on geographic region, the NTD risk was 1.39 (95% CI = 1.18-1.64) in Europe and 0.88 (95% CI 0.66-1.17) in the US. [29] The transatlantic difference may reflect

For peer review only

For peer review only - http://bmjopen.bmj.com/site/about/guidelines.xhtml

that the diet of smokers in Europe is less likely to be fortified with folate where fortification is voluntary and not mandatory. Analysis by NTD type also revealed a relationship between maternal smoking and spina bifida (OR 1.55 (95% CI = 1.06-2.26). [24] Therefore, inadequate dietary folate and decreased use of supplementation may contribute to the slight increased risk of NTDs in smokers. The results of our study demonstrated that current smokers were less likely than non-smokers to achieve the optimal RBC folate level of 906nmol/L associated with prevention of NTDs. Above this optimal RBC folate concentration, the risk of neural tube defects is reported to reduce to <8/10,000 live births. [30]

Women who continue to smoke in pregnancy may benefit from dietary intervention. A study of 862 women and infant pairs assessed maternal dietary quality at 24-28 weeks gestation. Lower dietary quality was associated with lower educational attainment, maternal smoking, obesity (pre-pregnancy) and a lack of exercise during pregnancy. It was found that increased dietary quality was associated with a reduced likelihood of a small for gestational age infant. Furthermore, a higher diet quality was positively associated with birth weight amongst former and current smokers. [31]

A study of two mother and infant cohorts (2461 pairs in Spain and 889 pairs in Greece) found that a high Mediterranean diet (MD - vegetables, legumes, fruit, nuts, cereals, seafood and fish and dairy products) adherence was associated with a lower risk of a growth restricted infant (risk ratio 0.5, 95% CI 0.3-0.9). When the analysis was stratified according to smoking status, higher MD adherence was associated with higher birth weight and birth length in the infants of mothers who continued to smoke. [32]

A strength of our study was that it was conducted prospectively and the study sample was well characterized. The detailed questionnaires on diet and supplements were supervised and analysis of this data considered up-to-date brand level information, including details on

For peer review only

food fortification. The information was collected in early pregnancy to minimize the recall bias associated with the completion of questionnaires after childbirth.

A limitation of the study was that convenience recruitment was used rather than consecutive recruitment which is not feasible in a busy maternity service with a single interviewer. The information on smoking was self-reported and not validated by biochemical testing. However, any non-disclosures of smoking were analysed with the non-smokers and thus, it is likely that the dietary differences we found would be even greater if smoking could be verified by biochemical screening. Dietary information was gathered in the first trimester only and it is not known if dietary choices changed as pregnancy advanced. An Irish study of 285 women who completed a three-day food diary in each trimester used cluster analysis to identify two major dietary patterns, "Unhealthy" and "Health conscious". Comparing the energy and nutrient intakes from the first trimester to the third trimester they found no significant change in either cluster. [33] It is also difficult to compare our findings on the relationship between dietary intakes and smoking behaviour in our study with previous reports because surveys on dietary intakes in adults usually excluded women who were pregnant or breastfeeding. [21]

For peer review only

CONCLUSIONS

Our findings that pregnant women who continue to smoke have dietary inadequacies strengthens the public health case for screening women in early pregnancy and for offering support for smoking cessation. The dietary intakes of former smokers suggest that dietary intakes improve after smoking cessation. For those women that continue to smoke in pregnancy, it also highlights the need to advise women about healthy eating behaviours and about the need to start Folic Acid supplementation.

ACKNOWLEDGEMENTS

This work was supported by the all–island body Safefood. We also thank the Friends of the Coombe for their support. We thank the women who participated and our laboratory and midwifery colleagues for their assistance.

AUTHOR CONTRIBUTIONS

EOM contributed to the conception and design of the study, collected data, performed data analysis and wrote and edited the manuscript. SC conducted the primary study involving patient recruitment, issuing questionnaires, analysis of food diaries and gathering data and contributed to the conception and design of this study and drafting of the manuscript. RK, CR and SC were involved in the conception and design of this study, data analysis and contributed to writing and editing. AM was involved in conception and design of the study, drafting sections of the manuscript and approving the final submitted version. MT contributed to the conception and design of the study, analysis of data and writing and editing of the manuscript.

For peer review

FUNDING

This work was supported by the all island body *Safe*food (grant number 02-2015).

DECLARATION OF INTERESTS

None of the authors have any conflicts of interest to declare.

PATIENT CONSENT

Written consent was obtained.

ETHICAL APPROVAL

The study was approved by the Coombe Women and Infants University Hospital Research and Ethics Committee (REC) – reference number 27-2013.

DATA SHARING STATEMENT

Extra data is available by emailing eimer.om@gmail.com.

For peer review only

REFERENCES

1. Shea AK, Steiner M. Cigarette smoking during pregnancy. *Nicotine Tob. Res*. 2008;10:267-278
2. Cnattingius S. The epidemiology of smoking during pregnancy: smoking prevalence, maternal characteristics, and pregnancy outcomes. *Nicotine Tob Res*, 2004;6:125-140
3. Blatt K, Moore E, Chen A, et al. Association of Reported Trimester-Specific Smoking Cessation and Fetal Growth Restriction. *Obstet Gynecol* 2015;125:1452-1459.
4. Barker DJP, Eriksson JG, Forsen T, Osmond C. Fetal origins of adult disease: strength of effects and biological basis. *Int J Epidemiol* 2002;31:1235-1239
5. Fall CH, Yajnik CS, Rao S, et al. Micronutrients and fetal growth. *J Nutr* 2003;133:1747-1756
6. Blumfield ML, Hure AJ, Macdonald-Wicks L, et al. Systematic review and meta-analysis of energy and macronutrient intakes in pregnancy in developed countries. *Nutr Rev* 2012;6:322-336
7. Fattah C, Farah N, O'Toole F, et al. Body Mass Index (BMI) in women booking for antenatal care: Comparison between selfreported and digital measurements. *Eur J Obstet Gynecol Reprod*. 2009;144:32–4.
8. Fattah, C, Farah, N, Barry, SC., et al. Maternal weight and body composition in the first trimester of pregnancy. *Acta Obstet Gynecol Scand* 2010;89(7):952-955.
9. McNulty B, Pentieva, K, Marshall, B, et al. Women's compliance with current folic acid recommendations and achievement of optimal vitamin status for preventing neural tube defects. *Hum Reprod*. 2011;26:1530-1536

For peer review only - http://bmjopen.bmj.com/site/about/guidelines.xhtml

10. Summary of dietary reference values – version 4 (September 2017) – European Food Safety Authority available at https://www.efsa.europa.eu/sites/default/files/2017_09_DRVs_summary_report.pdf Accessed on 21/2/2018
11. Goldberg GR, Black AE, Jebb SA, et al. Critical evaluation of energy intake data using fundamental principles of energy physiology: 1. Derivation of cut-off limits to identify under-recording. *Eur J Clin Nutr* 1991;45:569-581.
12. Black AE. Critical evaluation of energy intake using the Goldberg cut-off for energy intake: basal metabolic rate. A practical guide to its calculation, use and limitations. *Int J Obes Relat Metab Disord* 2000;24:1119-1130.
13. Central Statistics Office (CSO) (2013) Survey on income and living conditions, Cork, Ireland CSO. Available at: http://pdf.cso.ie/www/pdf/20170908023951_Survey_on_Income_and_Living_Conditions_2013_full.pdf
Accessed October 18, 2017
14. Coombe Women and Infants University Hospital Annual Clinical Report 2014. Available at: http://www.coombe.ie/index.php?nodeId=110. Accessed October 24, 2017
15. Lloveras G, Ribas LB, Ramon JM, Serra LM, Román BV. Food consumption and nutrient intake in relation to smoking. *Medicina clinica*. 2001;116(4):129-32.
16. Palaniappan U, Jacobs Starkey L, O'Loughlin J, et al. Fruit and vegetable consumption is lower and saturated fat intake is higher among Canadians reporting smoking. *J Nutr* 2001;131:1952-8.

17. Dyer AR, Elliot E, Stamler J, et al. (INTERMAP Research Group). Dietary intake in male and female smokers, ex-smokers, and never smokers: The INTERMAP Study. J *Hum Hypertens* 2003;17:641-54

18. Haibach JP, Homish GG, Giovino GA. A longitudinal evaluation of fruit and vegetable consumption and cigarette smoking. *Nicotine Tob Res*. 2012;15(2):355-63.

19. Cogswell ME, Weisberg P, Spong C. Cigarette smoking, alcohol use and adverse pregnancy outcomes: implications for micronutrient supplementation. *J Nutr*. 2003;133(5):1722S-1731S.

20. Mathews F, Yudkin P, Smith RF, et al. Nutrient intakes during pregnancy: the influence of smoking status and age. *J Epidemiol Community Health* 2000;54:17-23.

21. Subar AF, Harlan LC, Mattson ME. Food and nutrient intake differences between smokers and non-smokers in the US. *Am J Public Health* 1990;80:1323-9

22. Morabia A, Curtin F, Bernstein MS. Effects of smoking and smoking cessation on dietary habits of a Swiss urban population. *Eur J Clin Nutr*. 1999 Mar;53(3):239.

23. .Chelchowska M, Ambroszkiewicz J, Gajewska J, Laskowska-Kilta T and Leibschang J. The effect of tobacco smoking during pregnancy on plasma oxidant and antioxidant status in mother and newborn. *Eur J Obstet Gynecol Reprod Biol* 2011;155;132-136

24. Chéruel F, Jarleir M, Sancho-Garnier H. Effect of cigarette smoking on gustatory sensitivity, evaluation of the deficit and of recovery time course after smoking cessation. *Tob Induc Dis* 2017;15:15

25. King JC. Physiology of pregnancy and nutrient metabolism. *Am J Clin Nut* 2000;71:1218-1225.

For peer review only - http://bmjopen.bmj.com/site/about/guidelines.xhtml

26. Englund-Ogge L, Brantsaeter AL, Verena LS, et al. Maternal dietary patterns and preterm delivery: results from a large prospective cohort study. *BMJ* 2014;348:1446

27. Micronutrient needs during pregnancy and lactation. Drake VJ et al, updated 2016. Oregon state university. Report online. Available at: http://lpi.oregonstate.edu/mic/life-stages/pregnancy-lactation . Accessed on October 29th 2017

28. Mei-Dan E, Walfisch A, Weisz B, Hallak M, Brown R, Shrim A. The unborn smoker: association between smoking during pregnancy and adverse perinatal outcomes. *Journal of perinatal medicine*. 2015 ;43(5):553-8.

29. Wang M, Wang ZP, Gong R, et al. Maternal smoking during pregnancy and neural tube defects in offspring: a meta-analysis. *Childs Nerv Syst* 2013;30:83-89

30. Crider KS, Qi YP, Devin O, Tinker SC, Berry RJ. Modeling the impact of folic acid fortification and supplementation on red blood cell folate concentrations and predicted neural tube defect risk in the United States: have we reached optimal prevention? *Am J Clin Nutr.* 2018 https://doi.org/10.1093/ajcn/nqy065

31. Edmond JA, Karagas MR, Baker ER, et al. Better Diet Quality during Pregnancy Is Associated with a Reduced Likelihood of an Infant Born Small for Gestational Age: An Analysis of the Prospective New Hampshire Birth Cohort Study. *J Nutr.* 2018;148(1):22-30.

32. Chatzi L, Mendez M, Garcia R et al. Mediterranean diet adherence during pregnancy and fetal growth: INMA (Spain) and RHEA (Greece) mother-child cohort studies. *Br J Nutr*. 2012;107(1):135-45.

33. McGowan CA, McAuliffe FM. Maternal dietary patterns and associated nutrient intakes during each trimester of pregnancy. *Public Health Nutr*. 2013;16(1):97-107

For peer review only - http://bmjopen.bmj.com/site/about/guidelines.xhtml

For peer review only

Table 1. Comparison of characteristics between smokers and non-smokers with dietary data available (n=398)

	All women	Smokers	Non-smokers

	(n=398)[a]	(n=50)	(n=348)[b]	p
Age (years) (mean ± SD)	30.5 ± 5.6	27.0 ± 6.44	31.0 ± 5.3	<0.001
BMI (kg/m^2) (mean ± SD)	25.9 ±5.4	25.3 ± 4.7	26.0 ±5.5	0.418
Obese (%)	19.2% (76)	22% (11)	18.7% (65)	0.323
Nulliparous (%)	45.5% (181)	46.0% (23)	45.4% (158)	0.937
Irish-born (%)	76.9% (306)	82% (41)	76.1% (265)	0.739
Alcohol intake during pregnancy (%)	4.8 % (19)	6.1% (3)	4.7% (16)	0.863
Birth weight (grams) (mean ± SD)	3426.9 ± 586.1	3134.4 ± 622.1	3469.46 ± 569.2	<0.001

(significance level – 0.05, statistical tests used – chi-square test for categorical variables and independent samples t-test for continuous variables)

[a] Of the 398 women studied, delivery outcomes were available on 378; 330 who were non-smokers and 48 who were current smokers. The remaining women delivered elsewhere.

[b] As dietary composition was similar between never smokers and former smokers (Supplementary tables 1 and 2) they are combined into one "non-smokers" group.

FA – folic acid, SD – standard deviation, BMI – Body Mass Index

For peer review only

For peer review only

Table 2. Daily macronutrient and micronutrient intakes in smokers compared to non-smokers

	Smokers<br>(n=50)	Non-smokers<br>(n=348)[a]	p

	(median, IQ range)	(median, IQ range)	
Protein (g)	69.1 (31.3)	74.4 (28.0)	0.168
Carbohydrate (g)	204.3 (95.7)	198.8 (73.7)	0.820
Fat (g)	75.5 (42.8)	72.7 (39.1)	0.421
Saturates (g)	30.3 (15.1)	27.2 (15.7)	0.353
Monounsaturated fat (g)	25.3 (13.7)	24.5 (13.6)	0.928
Polyunsaturated fat (g)	8.8 (7.0)	10.3 (7.0)	0.176
Fibre (g)	13.1 (7.7)	16.3 (8.5)	<0.001
Sodium (mg)	2100.7 (1158.7)	1877.0 (971.9)	0.095
Potassium (mg)	2371.2 (1085.7)	2533.1 (1038.5)	0.305
Calcium (mg)	797.8 (538.06)	788.4 (536.4)	0.601
Magnesium (mg)	196.0 (75.8)	226.9 (100.5)	0.003
Phosphorous (mg)	1060.5 (381.7)	1180.8 (490.1)	0.063
Iron (mg)	7.9 (3.9)	9.5 (4.25)	0.002
Copper (mg)	0.7 (0.4)	0.9 (0.5)	0.001
Zinc (mg)	7.1 (3.9)	7.8 (3.7)	0.227
Chloride (mg)	3187.3 (1678.0)	2843.7 (1345.8)	0.067
Iodine (µg)	91.5 (89.5)	104.5 (77.0)	0.500
Retinol (µg)	312.6 (225.0)	282.9 (242.3)	0.916
Carotene (µg)	1488.3 (3427.4)	3213.2 (4479.5)	0.004
Vitamin D (µg)	2.3 (2.5)	2.4 (2.6)	0.547
Vitamin C (mg)	71.6 (74.3)	83.8 (83.0)	0.035
Vitamin E (µg)	7.1 (5.1)	8.2 (5.6)	0.196

For peer review only - http://bmjopen.bmj.com/site/about/guidelines.xhtml

Thiamine (mg)	1.3 (0.6)	1.4 (0.7)	0.100
Riboflavin (mg)	1.3 (0.9)	1.4 (0.7)	0.040
Niacin (mg)	30.5 (16.8)	34.1 (14.4)	0.089
Vitamin B6 (mg)	1.7 (1.0)	1.9 (0.9)	0.451
Vitamin B12 (µg)	4.0 (3.5)	4.0 (2.6)	0.767
Folate (natural and fortified) (µg)	202.1 (127.3)	241.9 (142.8)	0.006

(significance level – 0.05, statistical tests used; Kruskal-Wallis)

[a] As dietary composition was similar between never smokers and former smokers (Supplementary tables 1 and 2) they are combined into one "non-smokers" group.

SD – standard deviation, IQ range – Interquartile range, g – grams, mg – milligrams, µg - micrograms

For peer review only

Table 3. Total median (IQR) maternal dietary folate (including fortified folic acid) per day in early pregnancy and hematological measures of folate in non-smokers compared to current smokers (n=398).

	Non-smokers	Smokers	p

	(n=348)[a]	(n=50)	
Natural dietary folate intake (mcg)	193.9 (93.9)	174.1 (112.7)	0.029
Fortified dietary folate intake (mcg)	33.8 (50.7)	16.9 (59.4)	0.076
Total dietary folate intake (mcg)	241.9 (142.8)	202.1 (127.1)	0.006
Dietary folate equivalent (mcg)	268.2 (190.7)	214.6 (146.9)	0.005
Achieving WHO DFE for pregnancy (>/= 600 mcg DFE) (%, n)	6.3 % (22)	6.0 % (3)	0.930
Achieving WHO DFE for lactation (>/= 500 mcg DFE) (%,n)	10.9 % (38)	6.0 % (3)	0.285
Pre-pregnancy FA use (%,n)	48.0 % (167)	10.0 % (5)	0.015
Serum folate (nmol/L), (median, IQR)	35.2 +/- 17.0	22.3 +/- 19.5	<0.001
Red blood cell (RBC) folate (nmol/L), (mean, SD)	1195.1 +/- 440.0	820.0 +/- 362.5	<0.001
Achieving optimal RBC folate (%,n)[b]	71.3 % (248)	38.0 % (19)	<0.001

For peer review only - http://bmjopen.bmj.com/site/about/guidelines.xhtml

(significance level – 0.05, statistical tests used – chi-square test for categorical variables and independent samples t-test for continuous variables (normally distributed) and Kruskal-Wallis for comparison of non-normally distributed variables)

IQR– interquartile range, mcg – micrograms, WHO – World Health Organization, SD – standard deviation

DFE – Dietary folate equivalent. For natural folate from food – 1mcg folate from food is equal to 1mcg DFE. For food fortified with folate 1mcg of folate = 1.7 DFE, as natural folate from food is 50% bioavailable compared to 85% bioavailability from fortified folate and folate supplementation (85/50 = 1.7)

[a] As dietary composition was similar between never smokers and former smokers (Supplementary tables 1 and 2) they are combined into one "non-smokers" group.

[b] The reported optimal threshold concentration for red blood cell folate is reported as 906nmol/L by Daly et al.[30] At and above this concentration, the risk of neural tube defects (NTDs) was reduced to <8 NTDs/10,000 live births.

For peer review only

Supplementary Table 1:

Comparison of macronutrient intake between never smokers and former smokers

	Never smokers (n=193) (median (IQ range))	Former smokers (n=155) (median (IQ range))	P
Carbohydrate (g)	199.0 (68.2)	197.0 (89.6)	0.535
Protein (g)	72.8 (26.0)	77.0 (29.1)	0.027
Fat (g)	68.8 (40.1)	77.8 (36.4)	0.071
Saturated fat (g)	25.6 (14.2)	28.5 (16.3)	0.211
Monounsaturated fat (g)	24.0 (14.2)	25.5 (12.8)	0.256
Polyunsaturated fat (g)	10.1(7.2)	10.7 (7.3)	0.179
Fibre (g)	16.3 (8.8)	16.2 (8.0)	0.574

(Significance level, 0.05. Statistical test used – Kruskal-Wallis)

IQ range – interquartile range, g – grams

For peer review only

Supplementary Table 2: Comparison of micronutrient intake between never smokers and former smokers

	Never smokers (n=193) (median (IQ range))	Former smokers (n=155) (median (IQ range))	P
Sodium (mg)	1805.2 (928.7)	2069.6 (988.1)	0.008
Potassium (mg)	2472.9 (946.4)	2631.2 (1127.0)	0.102
Chloride (mg)	2688.6 (1294.4)	3067.6 (1329.7)	0.045
Calcium (mg)	749.0 (505.8)	838.0 (533.0)	0.120
Phosphorous (mg)	1124.5 (461.6)	1212.2 (463.5)	0.055
Iron (mg)	9.1 (3.5)	10.0 (5.5)	0.014
Magnesium (mg)	225.1 (98.0)	229.8 (110.3)	0.334
Zinc (mg)	7.7 (3.8)	8.1 (3.9)	0.041
Copper (mg)	0.9 (0.5)	0.9 (0.5)	0.501
Iodine (mcg)	101.8 (69.8)	106.9 (86.7)	0.080
Retinol (mcg)	274.6 (248.2)	310.9 (231.8)	0.089
Carotene (mcg)	3475.6 (5193.8)	2965.4 (4199.2)	0.300
Vitamin C (mg)	79.9 (88.5)	89.5 (78.2)	0.174
Vitamin D (mcg)	2.2 (2.5)	2.5 (2.9)	0.438
Vitamin E (mcg)	8.0 (5.5)	8.4 (5.5)	0.123

For peer review only

Thiamine B1 (mg)	1.4 (0.7)	1.4 (0.6)	0.293
Riboflavin B2 (mg)	1.4 (0.7)	1.6 (0.8)	0.008
Niacin B3 (mg)	33.3 (14.1)	35.8 (15.0)	0.038
Vitamin B6 (mg)	1.9 (0.8)	1.9 (0.8)	0.191
Vitamin B12 (mg)	3.8 (2.8)	4.1 (2.6)	0.018
Folic Acid (mcg)	234.1 (135.9)	249.0 (148.9)	0.154

(Significance level, 0.05. Statistical test used – Kruskal-Wallis)

IQ – interquartile range, mg – milligrams, mcg - micrograms

For peer review only

Supplementary Table 3: Comparison of macronutrient intakes between smokers and non-smokers (excluding likely energy under-reporters i.e. plausible reporters only)

	Non-smokers (n=222) (median (IQ range))[a]	Current smokers (n=34) (median (IQ range))	P
Carbohydrate (g)	217.3 (72.8)	230.0 (94.2)	0.229
Protein (g)	80.2 (25.1)	78.3 (33.5)	0.566
Fat (g)	84.3 (33.0)	90.6 (34.8)	0.416
Fibre (g)	18.3 (8.1)	14.6 (6.3)	0.004
Saturated fat (g)	31.5 (16.6)	35.5 (13.4)	0.472
Monounsaturated fat (g)	29.0 (12.5)	27.9 (12.8)	0.978
Polyunsaturated fat (g)	12.1 (7.3)	10.8 (8.3)	0.236

(Significance level, 0.05. Statistical test used – Kruskal-Wallis)

[a] As dietary composition was similar between never smokers and former smokers (Supplementary tables 1 and 2) they are combined into one "non-smokers" group.

IQ range – interquartile range, g - grams

For peer review only

Supplementary Table 4: Comparison of micronutrient intakes between smokers and non-smokers (excluding likely energy under-reporters i.e. plausible reporters only)

	Non-smokers (n=222) (median (IQ range))[a]	Current smokers (n=34) (median (IQ range))	P
Sodium (mg)	2126.2 (991.6)	2499.4 (1092.7)	0.079
Potassium (mg)	2785.8 (965.7)	2764.8 (1037.3)	0.631
Chloride (mg)	3156.8 (1399.0)	3624.4 (1747.9)	0.031
Calcium (mg)	961.9 (589.9)	848.8 (393.2)	0.396
Phosphorous (mg)	1299.2 (451.4)	1137.7 (479.4)	0.113
Iron (mg)	10.4 (4.3)	9.7 (3.7)	0.023
Magnesium (mg)	252.4 (101.0)	214.7 (80.8)	0.012
Zinc (mg)	8.6 (3.4)	8.1 (3.8)	0.357
Copper (mg)	1.0 (0.5)	0.8 (0.3)	0.009
Iodine (mcg)	120.5 (84.9)	124.8 (92.1)	0.889
Retinol (mcg)	344.9 (282.7)	344.2 (248.9)	0.836
Carotene (mcg)	3213.2 (4762.3)	1488.3 (3572.3)	0.016
Vitamin C (mg)	97.0 (90.8)	77.7 (69.6)	0.097
Vitamin D (mcg)	2.6 (2.9)	2.7 (2.5)	0.726
Vitamin E (mcg)	9.2 (5.4)	8.6 (7.2)	0.321

For peer review only

Thiamine B1 (mg)	1.5 (0.6)	1.5 (0.6)	0.486
Riboflavin B2 (mg)	1.7 (0.8)	1.5 (1.0)	0.244
Niacin B3 (mg)	36.1 (13.7)	33.4 (15.7)	0.562
Vitamin B6 (mg)	2.0 (0.8)	2.1 (0.8)	0.645
Vitamin B12 (mg)	4.5 (2.7)	5.1 (3.9)	0.528
Folic Acid (mcg)	266.2 (123.8)	213.4 (135.9)	0.009

(Significance level, 0.05. Statistical test used – Kruskal-Wallis)

[a] As dietary composition was similar between never smokers and former smokers (Supplementary tables 1 and 2) they are combined into one "non-smokers" group.

IQ range – interquartile range, mg – milligramsmcg – micrograms

For peer review only

STROBE 2007 (v4) checklist of items to be included in reports of observational studies in epidemiology*
Checklist for cohort, case-control, and cross-sectional studies (combined)

Section/Topic	Item #	Recommendation	Reported on page #
**Title and abstract**	1	(*a*) Indicate the study's design with a commonly used term in the title or the abstract	1
		(*b*) Provide in the abstract an informative and balanced summary of what was done and what was found	2
**Introduction**			
Background/rationale	2	Explain the scientific background and rationale for the investigation being reported	5
Objectives	3	State specific objectives, including any pre-specified hypotheses	5
**Methods**			
Study design	4	Present key elements of study design early in the paper	5-6
Setting	5	Describe the setting, locations, and relevant dates, including periods of recruitment, exposure, follow-up, and data collection	2,5-6
Participants	6	(*a*) *Cohort study*—Give the eligibility criteria, and the sources and methods of selection of participants. Describe methods of follow-up<br>*Case-control study*—Give the eligibility criteria, and the sources and methods of case ascertainment and control selection. Give the rationale for the choice of cases and controls<br>*Cross-sectional study*—Give the eligibility criteria, and the sources and methods of selection of participants	5-6
		(*b*) *Cohort study*—For matched studies, give matching criteria and number of exposed and unexposed<br>*Case-control study*—For matched studies, give matching criteria and the number of controls per case	n/a
Variables	7	Clearly define all outcomes, exposures, predictors, potential confounders, and effect modifiers. Give diagnostic criteria, if applicable	2
Data sources/ measurement	8*	For each variable of interest, give sources of data and details of methods of assessment (measurement). Describe comparability of assessment methods if there is more than one group	5-7
Bias	9	Describe any efforts to address potential sources of bias	6-7
Study size	10	Explain how the study size was arrived at	2
Quantitative variables	11	Explain how quantitative variables were handled in the analyses. If applicable, describe which groupings were chosen and why	6-7
Statistical methods	12	(*a*) Describe all statistical methods, including those used to control for confounding	7
		(*b*) Describe any methods used to examine subgroups and interactions	7
		(*c*) Explain how missing data were addressed	8
		(*d*) *Cohort study*—If applicable, explain how loss to follow-up was addressed<br>*Case-control study*—If applicable, explain how matching of cases and controls was addressed	n/a

For peer review only

		Cross-sectional study—If applicable, describe analytical methods taking account of sampling strategy	
		(*e*) Describe any sensitivity analyses	n/a
**Results**			
Participants	13*	(a) Report numbers of individuals at each stage of study—eg numbers potentially eligible, examined for eligibility, confirmed eligible, included in the study, completing follow-up, and analysed	8
		(b) Give reasons for non-participation at each stage	8
		(c) Consider use of a flow diagram	n/a
Descriptive data	14*	(a) Give characteristics of study participants (eg demographic, clinical, social) and information on exposures and potential confounders	8, 21-22
		(b) Indicate number of participants with missing data for each variable of interest	Footnotes of tables
		(c) *Cohort study*—Summarise follow-up time (eg, average and total amount)	n/a
Outcome data	15*	*Cohort study*—Report numbers of outcome events or summary measures over time	n/a
		Case-control study—Report numbers in each exposure category, or summary measures of exposure	n/a
		Cross-sectional study—Report numbers of outcome events or summary measures	24,25,26
Main results	16	(*a*) Give unadjusted estimates and, if applicable, confounder-adjusted estimates and their precision (eg, 95% confidence interval). Make clear which confounders were adjusted for and why they were included	n/a
		(*b*) Report category boundaries when continuous variables were categorized	27
		(*c*) If relevant, consider translating estimates of relative risk into absolute risk for a meaningful time period	n/a
Other analyses	17	Report other analyses done—eg analyses of subgroups and interactions, and sensitivity analyses	Supplementary tables
**Discussion**			
Key results	18	Summarise key results with reference to study objectives	8,9
Limitations	19	Discuss limitations of the study, taking into account sources of potential bias or imprecision. Discuss both direction and magnitude of any potential bias	14,14
Interpretation	20	Give a cautious overall interpretation of results considering objectives, limitations, multiplicity of analyses, results from similar studies, and other relevant evidence	10-14
Generalisability	21	Discuss the generalisability (external validity) of the study results	14
**Other information**			
Funding	22	Give the source of funding and the role of the funders for the present study and, if applicable, for the original study on which the present article is based	15,16

*Give information separately for cases and controls in case-control studies and, if applicable, for exposed and unexposed groups in cohort and cross-sectional studies.
Note: An Explanation and Elaboration article discusses each checklist item and gives methodological background and published examples of transparent reporting. The STROBE checklist is best used in conjunction with this article (freely available on the Web sites of PLoS Medicine at http://www.plosmedicine.org/, Annals of Internal Medicine at http://www.annals.org/, and Epidemiology at http://www.epidem.com/). Information on the STROBE Initiative is available at www.strobe-statement.org.

For peer review only
